# Disiloxanes and Functionalized Silica Gels: One Route, Two Complementary Outcomes—Guanidinium and Pyridinium Ion-Exchangers

**DOI:** 10.1371/journal.pone.0145680

**Published:** 2015-12-29

**Authors:** Łukasz Tabisz, Ainur Tukibayeva, Radoslaw Pankiewicz, Marta Dobielska, Boguslawa Leska

**Affiliations:** 1 Faculty of Chemistry, Adam Mickiewicz University in Poznań, Umultowska 89b, 61-614, Poznan, Poland; 2 Department of Chemistry, M. Auezov South Kazakhstan State University, Tauke Khan Ave 5, Shymkent, 160012, Kazakhstan; Kermanshah University of Medical Sciences, ISLAMIC REPUBLIC OF IRAN

## Abstract

Five novel disiloxane compounds comprising guanidinium and pyridinium moieties were obtained with high yields and purity. The verified synthetic pathways were then applied for modification of pre-functionalized silica gel, producing materials with the analogous organic side-chains. These halide-containing compounds and materials were then compared as to their ion-exchange properties: two disiloxanes proved to be effective in leaching different anions (nitrate, benzoate and ascorbate) from solid to organic phase, and pyridinium-functionalized silica gels showed selectivity towards perchlorate ion, removing it from methanolic solutions with preference to other singly charged anions. The results presented demonstrate that both compounds and materials containing silicon-carbon bonds can be produced using the same methodology, but offer strikingly different application opportunities. Comparison of their properties provides additional insight into the binding mode of different anions and hints at how the transition from a flexible siloxane bridge to immobilization on solid surface influences anion-binding selectivity. Additionally, one of the siloxane dipodands was found to form a crystalline and poorly soluble nitrate salt (1.316 g/L, water), although it was miscible with a wide range of solvents as a hydrochloride. A possible explanation is given with the help of semi-empirical calculations. A simple, time- and cost-efficient automated potentiometric titration methodology was used as a viable analytical tool for studying ion-exchange processes for both compounds and materials, in addition to standard NMR, FT-IR and ESI-MS methods.

## Introduction

Although silicon is carbon’s closest relative in the periodic table, the differences between these two elements are almost as abundant as the similarities. Organosilicon chemistry is based on the fact that although the energy of Si-O and Si-C bonds is similar to that of their purely organic counterparts, these bonds are longer and more polarized, which makes them more flexible in terms of physical and chemical properties [1–3].

The steric hindrance and, therefore, barrier for rotation is far lower in a siloxane backbone than in a carbon chain, which implies higher gas permeability, lower melting points and glass transition temperatures along with a wider range of solubility observed for siloxanes [4]. At the same time, the electronegativity of silicon atom (1.90, Pauling) is sufficiently low for the siloxane bridge to be cleaved in alkaline and highly acidic conditions [5]. Condensation of resulting silanol moieties, Si-OH, is the basis for obtaining a whole class of siliceous materials, from cost-effective amorphous silica gels, to specifically tailored silica nanoparticles (e.g. Stober silica)[6], to periodic mesoporous organosilicas (PMOs)[7]. All of those can be easily further functionalized. Taking advantage of the aforementioned reactivity of the polarized Si-O bond, one can graft specific organic moieties on the surface of a resilient, non-toxic and well-defined matrix, very often with a large surface area [8].

Silica-based materials are commonly used in the laboratory and frequently synthesized for purposes like separation [9], extraction [10], controlled drug release [11], templating [12], sensing [13], powder-type emulsions stabilization [14], heterogeneous catalysis [15] and many other. Siloxanes, however, are mainly encountered in polymer science, and the usefulness of smaller compounds comprising siloxane bridges remains fairly unexplored, with few notable exceptions in supramolecular chemistry of metal complexes [16–17]. Recently, we focused our attention on studying the differences between simple organic salts and silica gels with analogous, ionized moieties grafted on their surfaces; we concluded that ion-exchange selectivity can be well-defined even for uncomplicated ligands, and those tendencies can be further reinforced by preorganization of such ligands on rigid matrix [18–19]. Now, taking this approach a step further, we theorized that one synthetic pathway can lead to both novel disiloxane compounds and correspondingly functionalized siliceous materials, with potentially complementary properties. While hybrid, silica-based materials can be efficiently used in solid-phase extraction of hazardous compounds from solution [20], organosilicon compounds have been applied in phase-transfer catalysis [21] and are promising and versatile solubilizing agents.

**Fig 1:**
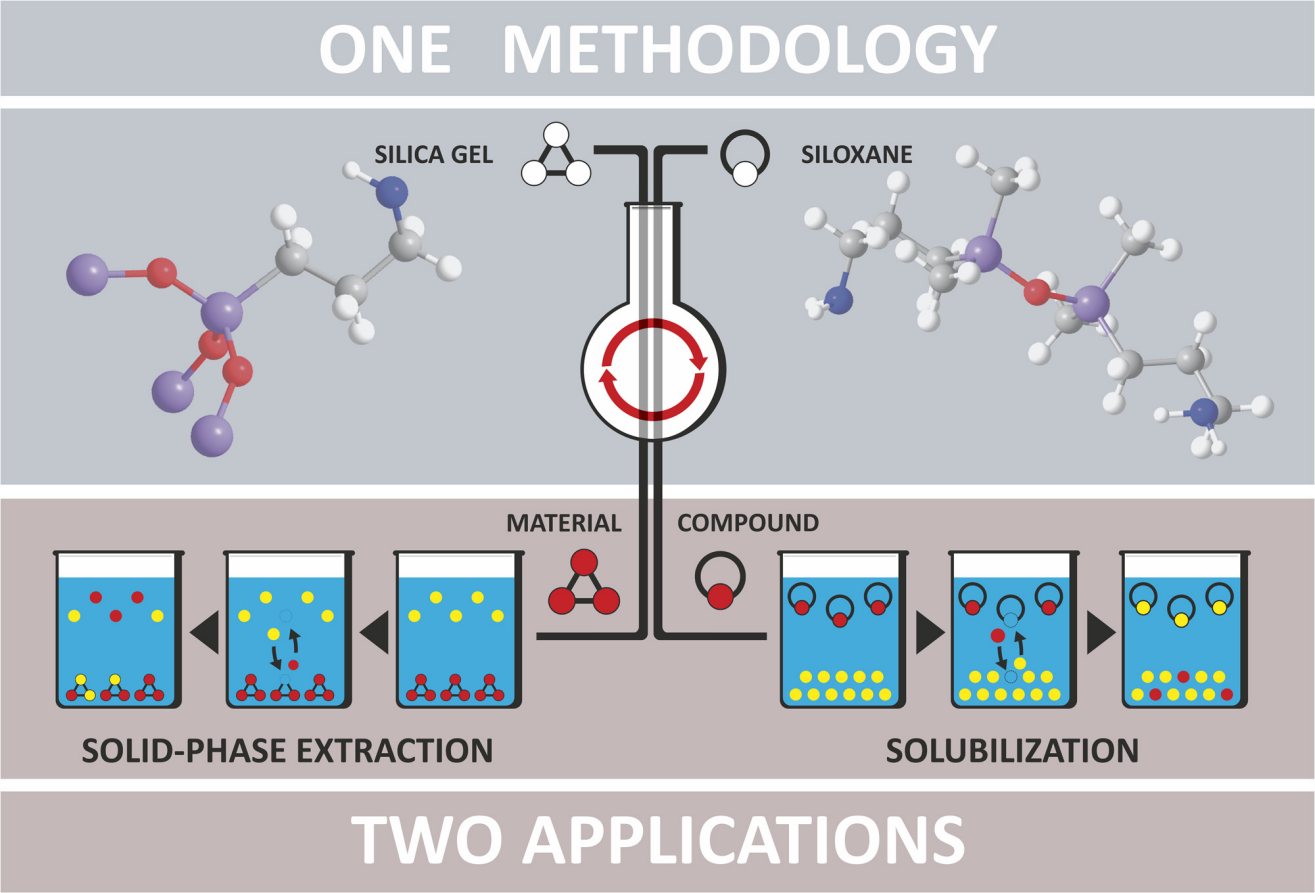
Visualization of the key research idea.

Two well-known organic functionalities, interesting in terms of anion-exchange, have been incorporated into (a) dipodal ligands with a siloxane bridge acting as the molecule’s center and (b) side chains of functionalized silica gels: guanidinium and pyridinium ionic groups. The former is renowned for the strong, doubly hydrogen-bonded system it forms with carboxylate ions (e.g. in peptides), as well as other oxo-anions (sulphate, phosphate) in some supramolecular complexes [22]. The latter enforces a more rigid structure on the molecule, which can be valuable in otherwise non-preorganized, podand-type receptors, and can form a variety of non-covalent bonds with anionic species, including pi-pi interactions and even weak hydrogen bonds, which incorporate the somewhat acidic protons in the para- and ortho- positions relative to the N^+^ atom in the pyridinium ring [23]. The resulting five novel disiloxane compounds and silica gels were employed in ion-exchange experiments to ascertain how the transition from a highly flexible molecule to rigid material affects the selectivity of anion recognition. The dualistic concept of the study presented is illustrated in Fig 1.

## Results and Discussion

### Synthesis and comparison of general properties

#### Outline

**Fig 2:**
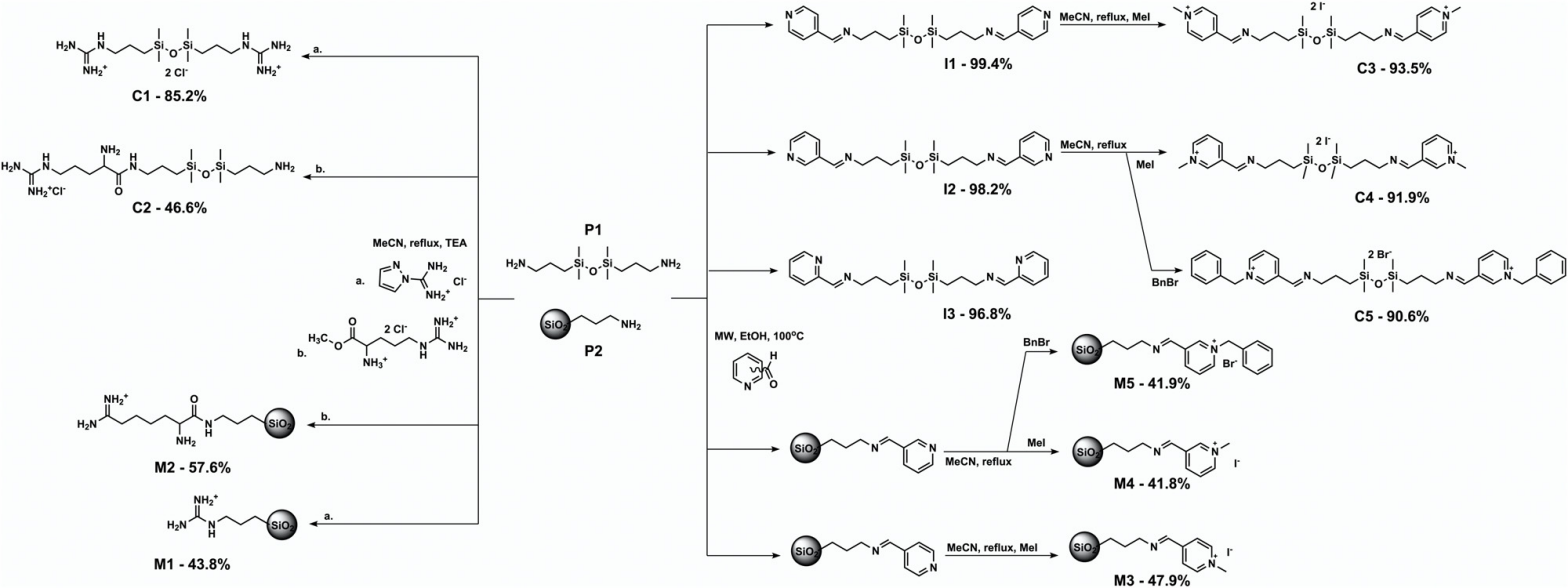
Overall synthetic scheme. MeCN—acetonitrile, TEA—triethylamine, MW—microwave-assisted synthesis, MeI—iodomethane, BnBr—benzyl bromide.

Different synthetic pathways were first investigated using the siloxane precursor; with highest possible yields and reduction of side reactions as our two main aims. Experimental conditions providing the best results were adapted for modification of pre-functionalized silica gel. This strategy proved to be effective in producing materials with satisfactory surface group conversion at the first attempt. However, it is worth noting that the above modification rates cannot be easily correlated with the yields of the corresponding organosilicon compounds; the former tend to fall in the 40–60% range regardless of the excess of reagent or time used in comparison with the synthesis of compound. The synthetic scheme showing yields/modification rates is presented as Fig 2.

#### Synthesis—remarks

The exclusion of moisture was crucial during the syntheses, especially for organosilicon compounds for which a “snowball effect” is observed, as minute amounts of reagent impurities cause disproportionally large amounts of side-products. With distilled and anhydrous substrates/solvents it was possible to acquire very pure compounds without the need to use chromatographic purification methods, which often prove ill-suited for siloxane compounds.

#### Microwave-assisted synthesis

It is worth noting that the reaction time when preparing Schiff bases **I1-I3** was cut down from 80 min with regular to just 10 min with microwave-assisted synthesis. The amount of solvent can also be sharply reduced with this method, resulting in much faster removal *in vacuo*, as the siloxanes can prove difficult to dry. The Schiff bases were obtained in yields close to 100% and were of excellent purity.

#### General properties

Compounds **C1-C5** were all resins, waxes or waxy solids (solidifying in temperatures close to ambient), difficult to dry and handle, unless lyophilization was performed; even those poorly soluble in water exhibited some degree of hygroscopicity. Only salt **C1** was found to be fully stable in the presence of moisture for extended periods of time, and solutions in D_2_O showed no changes in ^1^H NMR spectrum after one week. The pyridinium salts **C3-C5** turned out to be intensely colored compounds; their solutions showed only slight changes in ^1^H NMR spectra after 6h with samples that were intentionally wetted with deionized water prior to preparing the CD_3_CN solutions, but the salts started to decompose increasingly faster afterwards. This process was heralded by a gradual change in hue. For their hybrid material analogues **M3-M5**, however, the hydrolysis process was so rapid in aqueous solution that only traces of halide anions could be found in silica gel samples that were stirred for 15 min in deionized water. This increased instability verified the need to substitute it with anhydrous methanol in all ion-exchange experiments for materials **M3-M5**.

#### Solubility of compounds

As presumed, salts **C1-C5** demonstrated solubility in both polar and apolar media (Table 1). Interestingly, **C1-C2**, which separated from MeCN during the synthesis, were found to be perfectly soluble in chloroform and dichloromethane, which are less polar; at the same time it was observed that their more saturated solutions in those solvents appear viscous, almost gelatinous, while the same effect is not present in aqueous medium. Compounds **C3-C5** did not demonstrate similar solubility gap. Chloroform was chosen as the universally applicable solvent for ion-exchange experiments.

**Table 1:** Solubility of disiloxane products C1-C5.

Compound	Water	Ethanol	Acetone	Acetonitrile	Chloroform	Diethyl ether	Toluene
**C1**	VH	VH	L	VL	VH	N	N
**C2**	VH	VH	A	L	H	N	N
**C3**	A	H	H	H	A	N	N
**C4**	L	H	H	H	A	N	N
**C5**	VL	A	A	A	H	N	N^a^

VH > 0.5 mmol/mL > H > 25 μmol/mL > A > 10 μmol/mL > L > 1 μmol/mL > VL; N—insoluble.

^a^There was some very weak coloring of the toluene layer, observed for this compound only.

#### Characterization of materials

**Fig 3:**
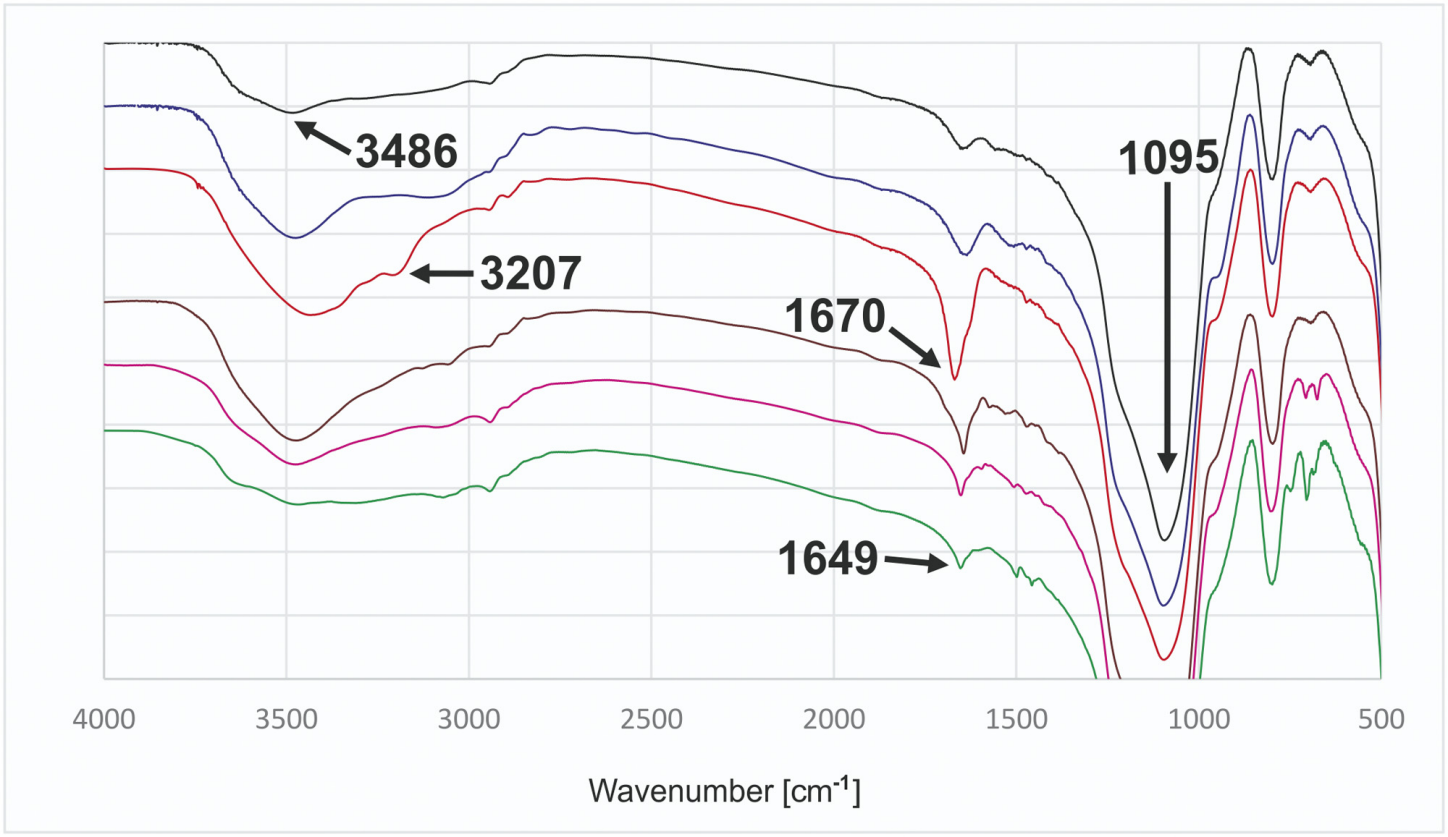
FT-IR spectra of hybrid siliceous materials (stacked). **P2** (black), **M1** (blue), **M2** (red), **M3** (brown), **M4** (magenta), **M5** (green).

Apart from potentiometric titration experiments providing quantitative information about modification of silica gels (Table 2), infrared spectra were recorded for the precursor and resulting hybrid materials to further demonstrate that the intended modification has occurred. In all spectra (Fig 3) we can observe a dominant band with a maximum at about 1095 cm^-^, which is characteristic of silica materials and assigned to the ν(Si-O) vibrations. All the spectra show signals assigned to the ν(N-H) or ν(+N-H) stretching vibrations as broad bands at about 3500 cm^-1^. In the spectra of modified hybrid materials there is a characteristic band with a maximum at 1649 cm^-1^ assigned to ν(C = N) stretching vibrations of imine groups. The spectrum of material **M2** can be discerned by the presence of bands with maxima at 1670 cm^-1^ and about 3200 cm^-1^, assigned to the amide group stretching vibrations, ν(C = N) and ν(N-H), respectively.

**Table 2:** Modification parameters for materials M1-M5.

Material	Modification rate [%]	Loading A^a^ [mmol/g]	Loading B^b^ [mmol/g]	Material color
**M1**	43.8	0.71	0.92	off-white
**M2**	57.6	0.84	0.62	pale yellow
**M3**	47.9	0.70	0.76	orange
**M4**	41.8	0.62	0.86	intense yellow
**M5**	41.9	0.61	0.85	dark red

Values were calculated on the basis of precursor material specifications and automated titration experiments (halide content). For details see Materials and Methods —**Potentiometric titrations—Modification rates**.

^a^Loading of modified, ionic groups (as shown in Fig 2).

^b^Loading of leftover, unmodified propylamine groups (SiO_2_-CH_2_-CH_2_-CH_2_-NH_2_).

### Ion-exchange experiments

#### Comparability

No single method can be applied to both materials and compounds and provide directly comparable, quantitative information about their ion-exchange properties. However, as we have shown before, certain tendencies evidenced by compounds in their selective extraction of ions (from the solid phase to the apolar solvent) can indeed be transferrable to materials bearing similar organic moieties (used in extraction of those ions from a polar medium to the solid phase)[18–19]. This is especially interesting since the change of solvent has been known to dramatically impact both the effectiveness and selectivity of a molecular receptor. Analyzing similarities and differences between mobile and immobilized functions can provide clues as to whether the flexibility of a molecule plays a part in selective binding of anions and how great is the impact of non-specific, ion-ion interactions.

### Disiloxanes: solubilization of anions

#### Overview

In general, the compounds examined exhibited very different affinities towards counter-ions (Table 3). The amount of substituted halide anions varied from nearly 0% to 85%; the two most effective exchangers/solubilizers were compounds **C1**—selective for nitrate—and **C3**—selective for benzoate (and, to a lesser extent, ascorbate). The discrepancies observed prove that the solubility of salts used in similar experiments affect the results only to a minor extent, and they do not overshadow factors like host-guest complementarity. This in turn suggests strongly that the counter-ion substitution occurs mostly at the solid-liquid interface.

**Table 3:** Ion-exchange properties of disiloxanes C1-C5.

Compound(as halide)	Acetate	Benzoate	Ascorbate	Oxalate	Nitrate	Sulphate
**C1**	0.32	0.18	0.47	0.31	0.85	0.46
**C2**	0.53	0.32	0.27	0.27	0.48	0.16
**C3**	0.14	0.07	0.03	-	<0.01	0.03
**C4**	0.32	0.77	0.66	-	0.36	0.03
**C5**	<0.01	0.08	0.03	-	0.03	0.01

Results are presented as molar fractions of halide ions exchanged for the anion from the solid phase. For details see Materials and Methods—**Potentiometric titrations—Ion-exchange: disiloxanes**.

#### Guanidinium receptors

While compound **C2** exhibited moderate exchange rates, with foreseeable preference for acetate and nitrate anions, compound **C1** showed remarkable affinity for nitrate and unexpectedly high one for ascorbate. These differences, along with peculiar solubility mentioned earlier led us to believe that the smaller (but more symmetric) molecules of **C1** prefer to form supramolecular structures stabilized by certain counter-ions more efficiently than others. Low values for oxalate and sulfate—despite their bivalent character—can be explained either by the anions’ larger size and inability to “hide” their charge efficiently inside such structures, or the difficulty in separating a doubly-charged ion from its crystal lattice.

#### Pyridinium receptors

Only compound **C4** showed some potential in transferring the selected anions to the organic phase; surprisingly, its isomer **C3** behaved in the experiments more like compound **C5**. One possible explanation is the higher symmetry (easier organization) of **C3** and more sterically hindered structure of **C5**, in the case of positively charged atom in a non-terminal position, both factors could be inhibiting the molecule’s ability to disrupt the crystal lattice.

**Fig 4:**
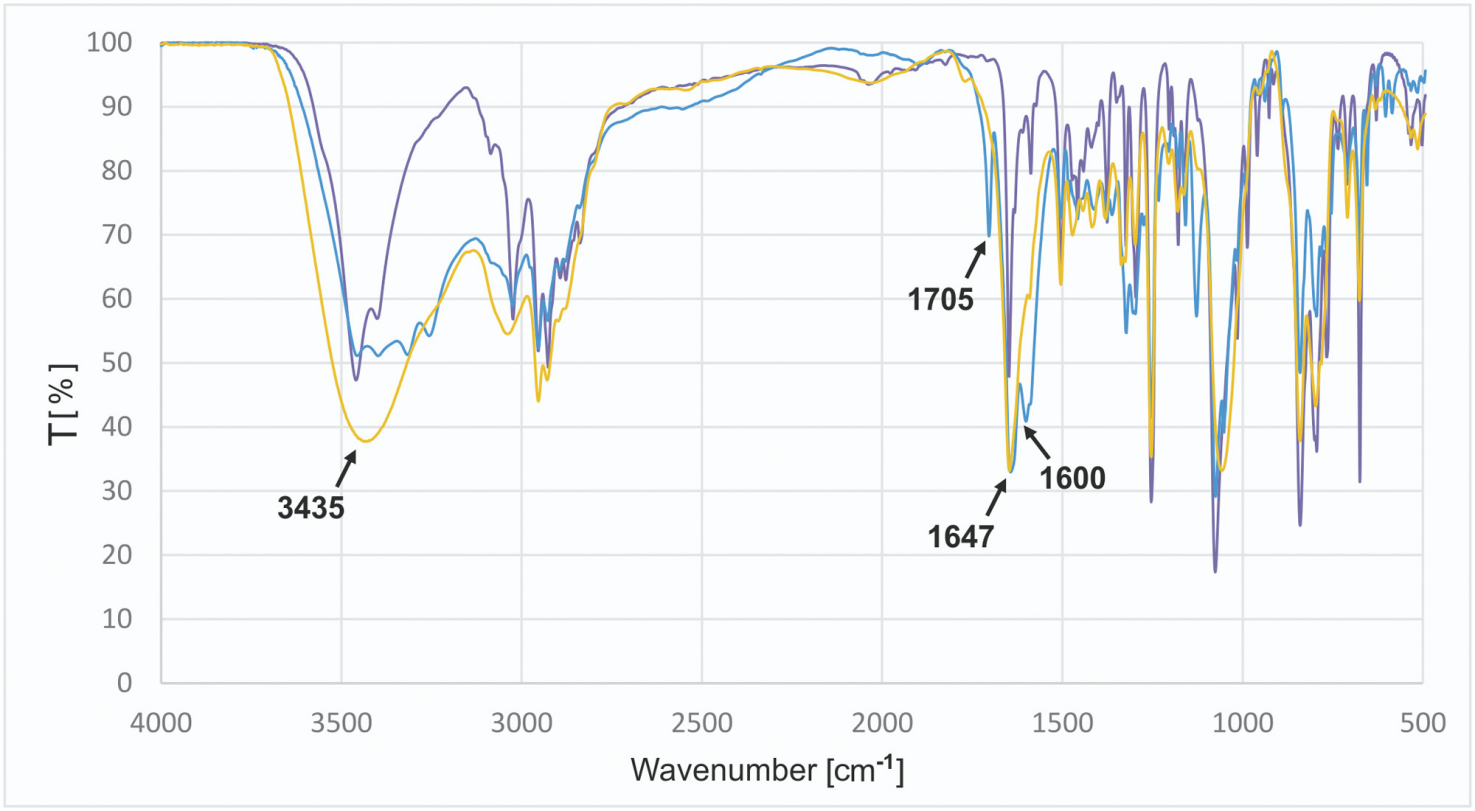
FT-IR spectra evidence for selective binding of ascorbate by C4 (superimposition). Pure iodide salt of **C4** (violet), iodide salt of **C4** mixed with sodium ascorbate in 1:2 molar ratio (blue), ascorbate salt of **C4** (yellow).

Preferences of compound **C4** were somewhat complementary to those of guanidinium receptors: larger and less symmetrical benzoate and ascorbate were leached selectively. Because sodium ascorbate is virtually insoluble even in methanol, not to mention less polar solvents, we were interested in how this particular—evidently strong—binding occurs. Infrared spectra of three **C4** samples in KBr were recorded: as a pure iodide salt, mixed with sodium ascorbate (in 1:2 molar proportion, ground separately) and after equilibration with a 10-fold molar excess of sodium ascorbate (Fig 4). The sharp signals with maxima in range 3200 cm^-1^–3600 cm^-1^, assigned to ν(O-H) stretching vibrations of ascorbate ion, disappear after equilibration. Instead, a strong, broad band with a maximum at 3435 cm^-1^ appears. This indicates the presence of only one type of oxygen-hydrogen bonds, of the same strength. The changes were also visible in the lower range, where the band assigned to ν(C = O) vibrations with a maximum at 1647 cm^-1^ was clearly strengthened—at the cost of bands with maxima at 1705 cm^-1^ and 1600 cm^-1^ disappearing altogether. These observations confirm the strong interactions between **C4** and ascorbate.

### Silica gels: solid-phase extraction of anions

#### Overview

In contrast with their compound analogues, materials bearing one type of ionic group showed similar tendencies towards the anions examined (Table 4). At the same time, bivalent ions were strongly favored over monovalent ones in the solid-phase extraction processes studied. It can be easily explained by accumulation of positive charge on the modified silica surface, which amplifies the impact of ion-ion electrostatic interactions. These observations further substantiate the claim that siloxanes **C1-C5** discriminate between counter-ions based on their intrinsic and/or supramolecular structure in solution, which is an ability that is mostly lost after grafting on a solid support. Sodium perchlorate was chosen as a replacement for salts that were insufficiently soluble in methanol to perform ion-exchange experiments for materials **M3-M5**, with surprising results.

**Table 4:** Ion-exchange properties of materials M1-M5.

Material	Bound anion (B)	Free anion (F)							
		Halide	Acetate	Benzoate	Ascorbate	Oxalate	Nitrate	Sulphate	Perchlorate
**M1**	**Halide**	1	1.36E+00	7.91E-01	1.22E+00	1.63E+03	2.76E+00	1.59E+03	-
**M2**	Halide	1	2.14E+00	3.70E+00	1.32E+00	6.19E+03	1.10E+01	3.08E+03	-
**M3**	Halide	1	4.50E-01	1.07E+00	-	-	2.35E+00	-	2.16E+01
**M4**	Halide	1	9.49E-01	9.64E-01	-	-	1.67E+00	-	1.53E+01
**M5**	Halide	1	2.20E-01	3.16E-01	-	-	1.52E+00	-	9.15E+00
**M1**	**Acetate**	7.35E-01	1	5.81E-01	8.99E-01	8.80E+02	2.02E+00	8.59E+02	-
**M2**	Acetate	4.67E-01	1	1.73E+00	6.17E-01	1.35E+03	5.14E+00	6.72E+02	-
**M3**	Acetate	2.22E+00	1	2.37E+00	-	-	5.23E+00	-	4.79E+01
**M4**	Acetate	1.05E+00	1	1.02E+00	-	-	1.76E+00	-	1.61E+01
**M5**	Acetate	4.55E+00	1	1.44E+00	-	-	6.89E+00	-	4.16E+01
**M1**	**Benzoate**	1.26E+00	1.72E+00	1	1.55E+00	2.60E+03	3.48E+00	2.54E+03	-
**M2**	Benzoate	2.70E-01	5.78E-01	1	3.57E-01	4.52E+02	2.97E+00	2.25E+02	-
**M3**	Benzoate	9.39E-01	4.23E-01	1	-	-	2.21E+00	-	2.03E+01
**M4**	Benzoate	1.04E+00	9.84E-01	1	-	-	1.73E+00	-	1.58E+01
**M5**	Benzoate	3.16E+00	6.96E-01	1	-	-	4.80E+00	-	2.89E+01
**M1**	**Ascorbate**	8.17E-01	1.11E+00	6.46E-01	1	1.09E+03	1.84E+00	1.30E+03	-
**M2**	Ascorbate	7.58E-01	1.62E+00	2.80E+00	1	3.55E+03	6.31E+00	2.33E+03	-
**M1**	**Oxalate**	6.14E-04	1.14E-03	3.84E-04	9.20E-04	1	4.66E-03	9.77E-01	-
**M2**	Oxalate	1.61E-04	7.39E-04	2.21E-03	2.81E-04	1	1.95E-02	4.97E-01	-
**M1**	**Nitrate**	3.63E-01	4.94E-01	2.87E-01	4.44E-01	2.15E+02	1	2.10E+02	-
**M2**	Nitrate	9.10E-02	1.95E-01	3.37E-01	1.20E-01	5.13E+01	1	2.55E+01	-
**M3**	Nitrate	4.25E-01	1.91E-01	4.53E-01	-	-	1	-	9.17E+00
**M4**	Nitrate	5.99E-01	5.68E-01	5.77E-01	-	-	1	-	9.14E+00
**M5**	Nitrate	6.60E-01	1.45E-01	2.08E-01	-	-	1	-	6.03E+00
**M1**	**Sulphate**	6.28E-04	1.16E-03	3.93E-04	9.41E-04	1.02E+00	4.77E-03	1	-
**M2**	Sulphate	3.25E-04	1.49E-03	4.45E-03	5.66E-04	2.01E+00	3.92E-02	1	-
**M3**	**Perchlorate**	4.64E-02	2.09E-02	4.94E-02	-	-	1.09E-01	-	1
**M4**	Perchlorate	6.55E-02	6.22E-02	6.31E-02	-	-	1.09E-01	-	1
**M5**	Perchlorate	1.09E-01	2.41E-02	3.46E-02	-	-	1.66E-01	-	1

Results are presented as values of selectivity coefficients, ***k***_***B/F***_. For further details see Materials and Methods—**Potentiometric titrations—Ion-exchange: materials**.

#### Guanidinium-comprising materials

Minor differences in the ion-exchange profiles of **M1** and **M2** can be attributed to guanidinium moieties being placed very close to the surface (and unmodified propylamine groups) in the former, and at a more distant position in the latter, which grants even easier approach for larger anions. Low selectivity coefficients for nitrate suggest that the forced ordering of protonated guanidines near the silica surface precludes their geometrical arrangement that is responsible for the selective binding of this anion in solution.

#### Pyridinium-comprising materials

**Fig 5:**
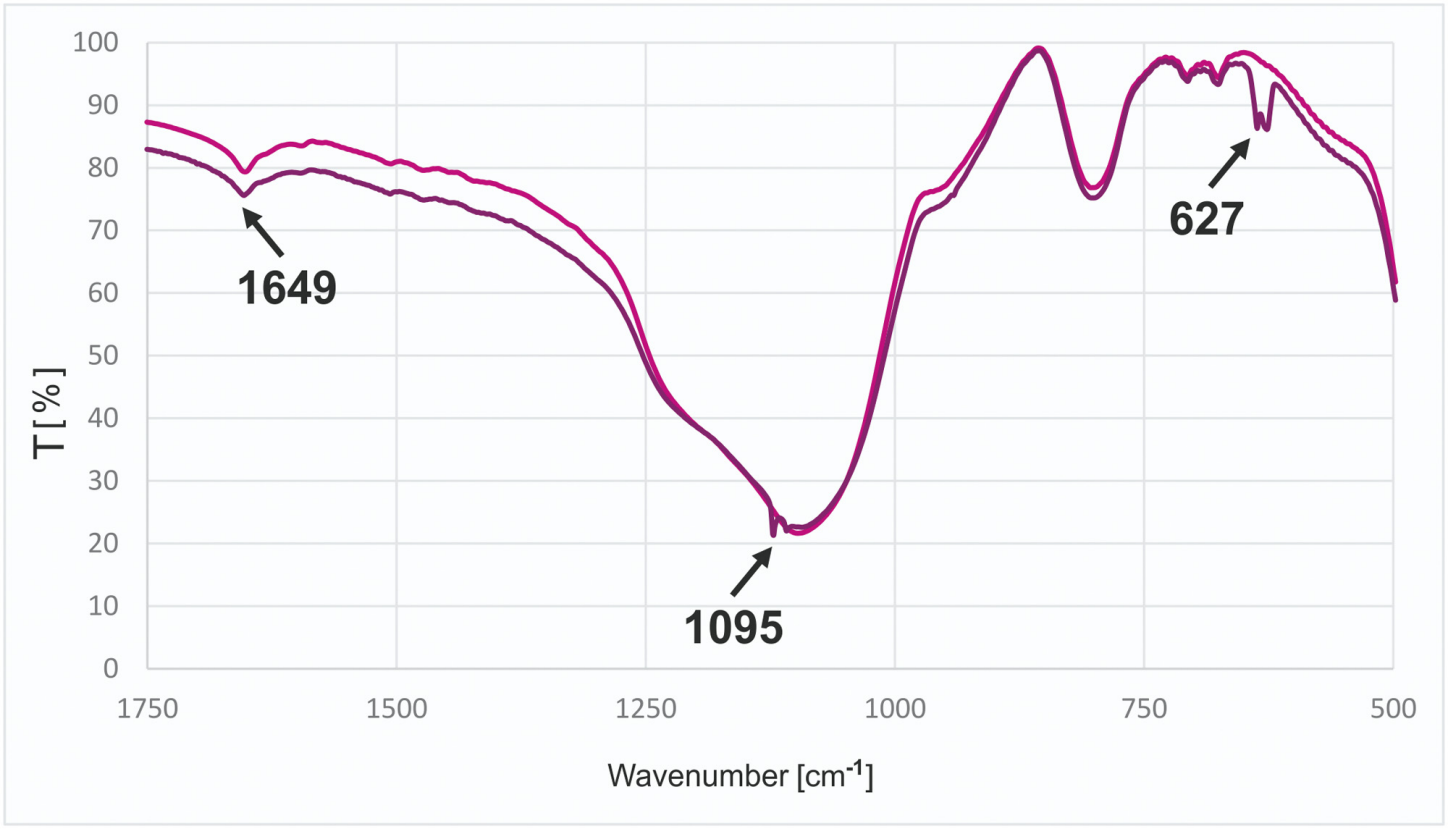
FT-IR spectra of M4 before and after extraction of perchlorates (superimposition). Pure, iodide form of **M4** (magenta), sample after equilibration in methanolic solution of sodium perchlorate (violet).

The discrepancies observed for disiloxanes were barely noticeable for their material counterparts, as was the selectivity shown by **C4** towards benzoate. Instead, reluctance of **M5** to liberate bromide can be seen as a sign of either a moderate selectivity towards the halide, or the benzyl groups shielding the positively-charged nitrogen, weakening its possible interaction with other anions in solution. We were, however, more surprised to find the perchlorate ion to be distinct from other anions examined, and selectively bound by **M3-M5**, roughly by an order of magnitude (values of selectivity coefficients). Perchlorate ion is commonly used in supramolecular chemistry as a non-interfering counter-ion when cation receptors are examined [24], and to substantiate the claim that it is preferably extracted from solution in this particular case, and FT-IR spectrum of **M4** after equilibration in sodium perchlorate solution was recorded. When compared with the spectrum before the exchange (Fig 5), the only apparent difference was the appearance of a signal with a maximum at 627 cm^-1^. The other characteristic signal assigned to perchlorates, much more intense, with a maximum at about 1100 cm^-1^, was concealed by the very strong signal assigned to the ν(Si-O) vibrations of the silica matrix.

### 1,3-Bis-(3-guanidylpropyl)-1,1,3,3-tetramethyldisiloxane dihydrochloride as a selective nitrate receptor

#### Overview

The most apparent case of selectivity in anion-exchange encountered during the course of presented work was the binding of nitrate by a rather simple, dipodal compound **C1**. To establish whether the preference would also be seen in the highly competitive, aqueous medium we decided to perform NMR titrations. However, we soon discovered that while the hydrochloride salt (light-yellow resin) was fully miscible with water, the addition of nitrate to the solution resulted in clouding and appearance of white precipitate. This was quite a new phenomenon to us, as none of the siloxane compounds we have worked with before precipitated so readily, usually producing amorphous solids from concentrated solutions only after a long periods of time, or slowly solidifying after all solvent traces were successfully removed. Crystallization of a siloxane compound has been achieved for some Schiff base—metal complexes, however [25]. Interestingly, there was no difference between the ^1^H NMR spectra of chloride and nitrate salts.

#### Solubility and selectivity

The solubility of **C1** as a nitrate salt in water was found to be a very moderate 1.316 g/L (+/-3%) at 30°C. This corresponds to the concentration of a saturated solution of 0.0294 mol/L and its density of 1.03 g/L. Assuming that the precipitate is a simple salt and in aqueous solution it undergoes full dissociation, the solubility product can also be calculated:

$$\begin{array}{l} \left[\left(C1\right){\left(NO_{3}\right)}_{2}\right]↔C1^{2+}+2NO_{3}^{-}\\ K_{sp}=1.01*10^{-4} \end{array}$$

In similar quick tests as that with sodium nitrate, cloudiness and precipitation in disodium sulfate solution appeared only at concentrations more than three times higher, and with sodium phosphate (monobasic), they did not appear at all (up to 1 mol/L).

#### Elemental analysis

**Fig 6:**
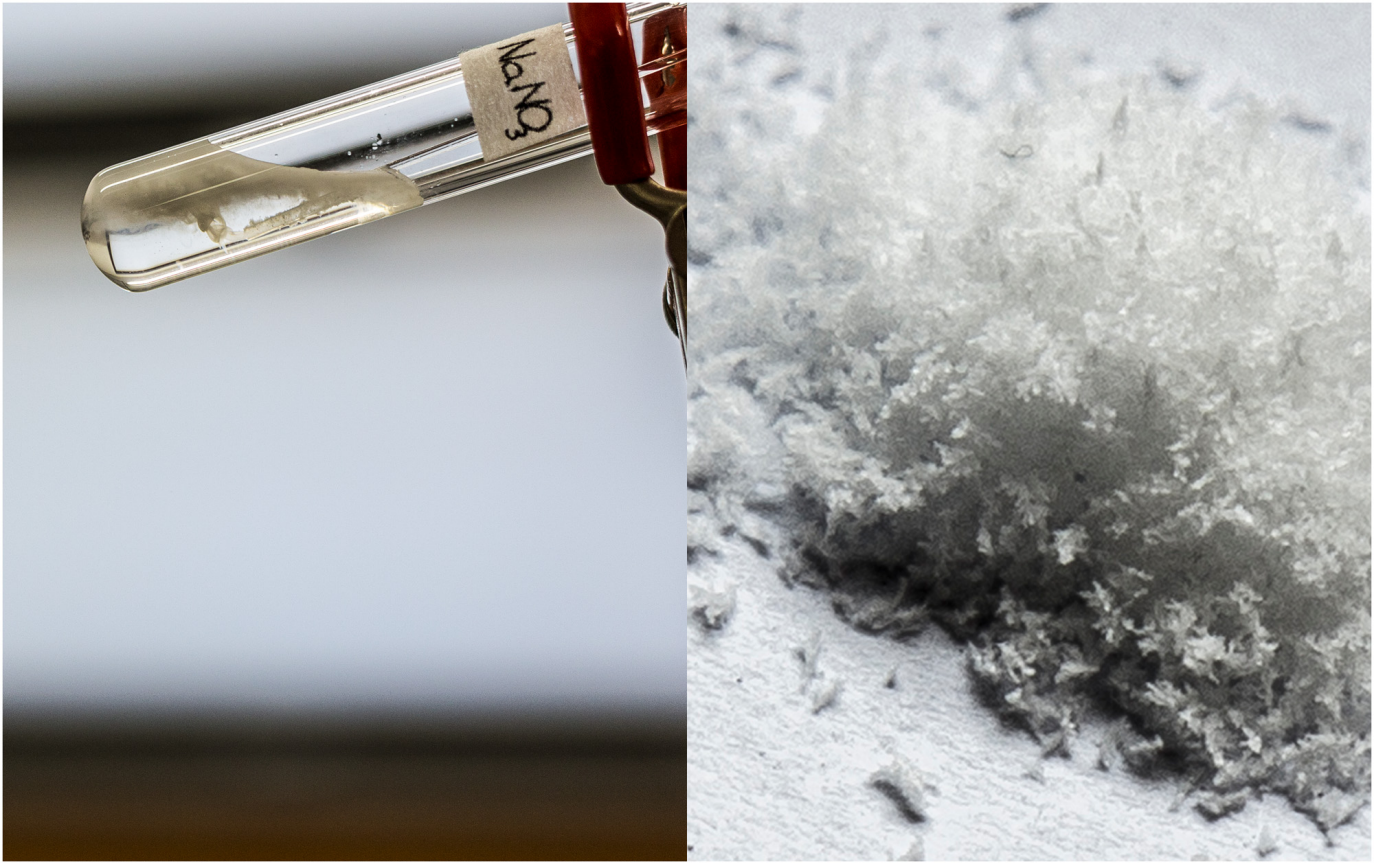
Nitrate salt of compound C1. Immediate precipitation after addition of **C1** to aqueous NaNO_3_ solution (left) and the recrystallized form (right).

To confirm the composition of the precipitate, elemental analysis was performed for two samples: **C1** as the synthesized, chloride salt, and precipitate from sodium nitrate solution, before and after recrystallization from water-ethanol mixture (5:1, v:v) that was allowed to slowly evaporate during the course of ten days. Small, white, sprig-shaped crystals (Fig 6) were successfully obtained in this fashion instead of amorphous, slightly waxy solid (m.p. 155.7–157.4°C). Experimental and calculated values are gathered in Table 5.

**Table 5:** Elemental analysis for compound C1 isolated as chloride and nitrate salt.

Salt (C1)	Chloride			Nitrate		
Element	C	H	N	C	H	N
**Calculated [%]**	35.54	8.45	20.72	31.43	7.47	24.43
**Found [%]**	35.44	8.55	20.28	29.38^a^/30.77^b^	6.77^a^/7.59^b^	23.01^a^/24.91^b^

^a^ crude precipitate, ground and dried

^b^ recrystallized, ground and dried.

#### Infrared spectra

**Fig 7:**
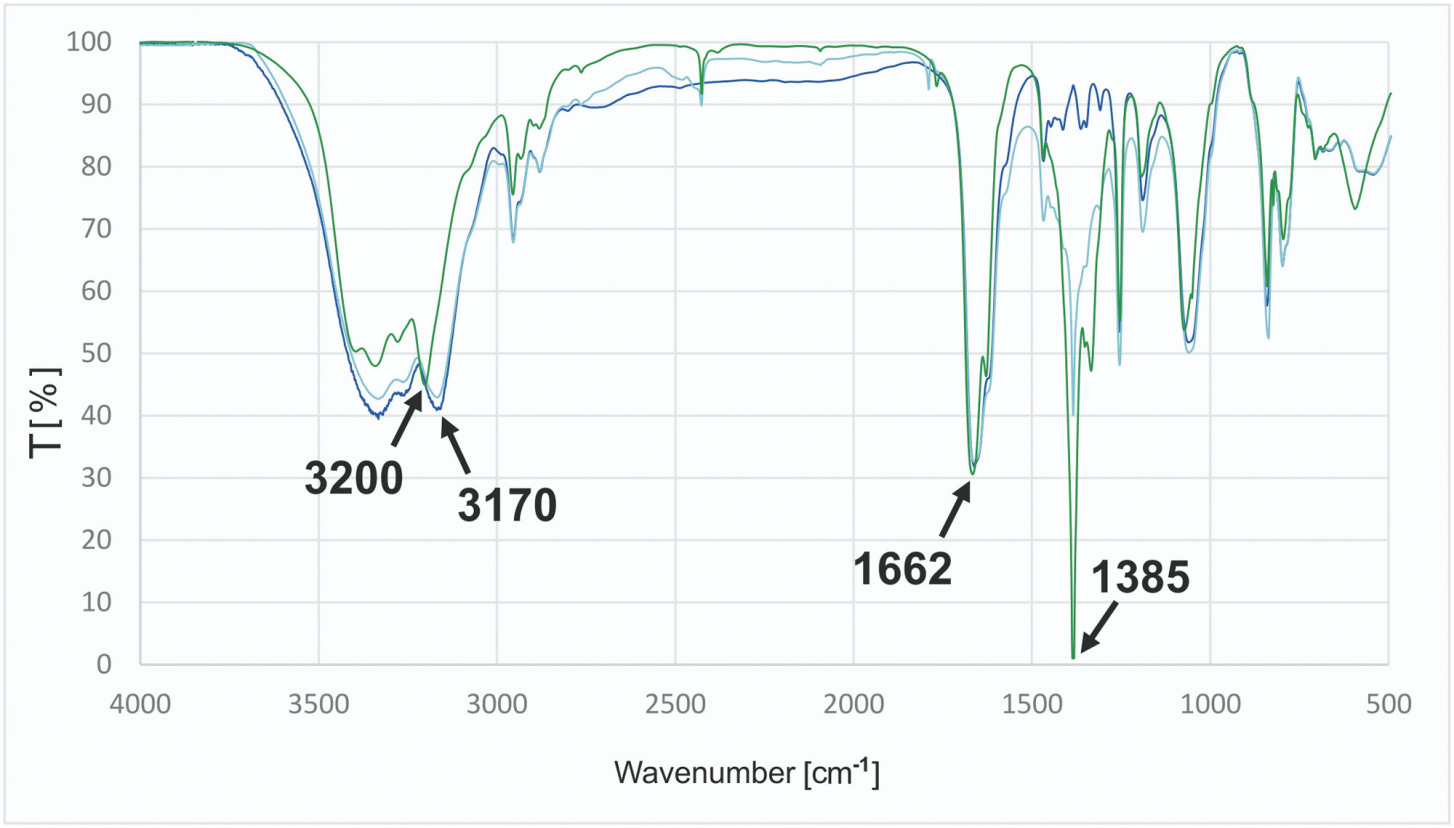
FT-IR spectra evidence for selective binding of nitrate by C1 (superimposition). Chloride salt of **C1** (navy blue), chloride salt of **C1** mixed with sodium nitrate in 1:2 molar ratio (pale blue), nitrate salt of **C1** (green).

As ^1^H NMR spectra failed to show important changes during the complexation of nitrate by compound **C1**, FT-IR spectra of three samples in KBr were recorded: the chloride salt, chloride salt mixed with sodium nitrate (1:2 molar ratio, ground separately) and the nitrate salt. Their superimposition is presented in Fig 7. Despite the lack of change in the position or intensity of the band with a maximum at 1662 cm^-1^ (assigned to the ν(C = N) vibrations of guanidine groups), the signal at 1385 cm^-1^ (assigned to ν(N = O) vibrations of nitrate) was greatly amplified in the spectrum of nitrate salt of **C1**. compared to that observed for the mixture. More interesting situation can be observed in the range 3100 cm^-1^–3500 cm^-1^. The maximum of the band assigned to the ν(N-H) vibration of secondary amino group shifts from 3170 cm^-1^ to 3200 cm^-1^, reflecting the strengthening of N—H bond. The appearance of another maximum of absorption also suggests more complicated interactions between the guanidine group and nitrate than with chloride.

#### Theoretical calculations

**Fig 8:**
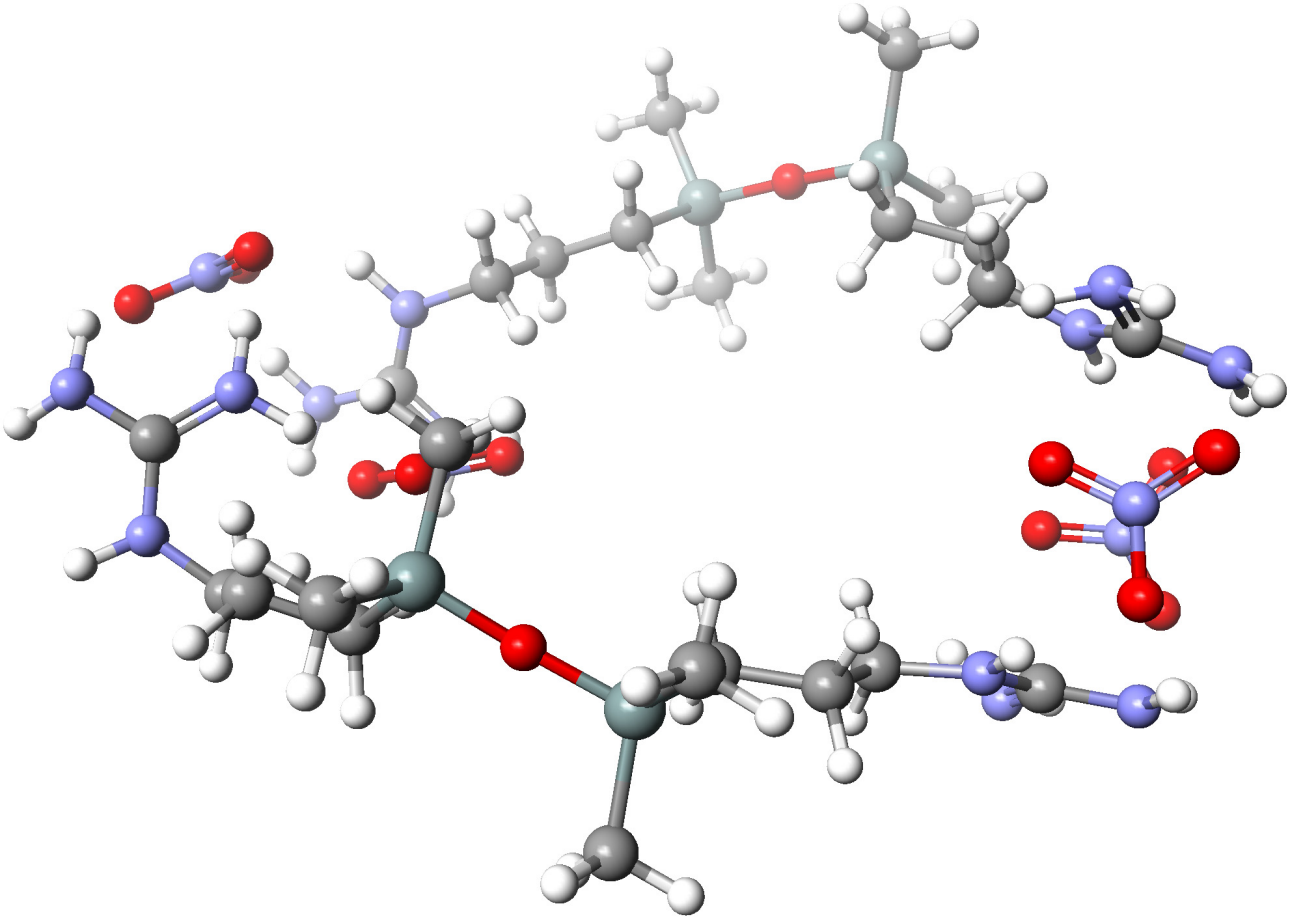
Calculated (PM6) structure of the energetically favorable dimeric nitrate salt of C1.

One possible explanation for the unusual interaction between **C1** and nitrate ions could be the formation of a larger and more rigid supramolecular aggregate. To visualize the two most probable structures of a **C1**-nitrate complex: a monomer (1:2) and dimer (2:4), semi-empirical calculations were carried out using the parametric method 6 (PM6). The calculations show that the formation of a dimeric entity is greatly energetically favorable: the delta of heat of complex formation (ΔHOF) for a dimer is lower by 226.4 kJ/mol than the sum of (ΔHOF) of two individual monomers. The calculated structure of the (2:4) complex is presented in Fig 8.

## Materials and Methods

### Precursors and reagents

#### Synthesis

1,3-Bis-(aminopropyl)-1,1,3,3-tetramethyldisiloxane (**P1**) was obtained from ABCR. Precursor material (propylamine-functionalized silica gel, **P2**) was obtained from SiliCycle Inc. (“SiliaBond^®^ Amine”–see S1 Table for specifications). Other reagents and building blocks were obtained from Sigma-Aldrich. Whenever applicable, purification by means of low-pressure distillation was performed. Solvents were obtained from POCH Corp. and were of analytical grade or higher. All were subjected to drying over molecular sieves.

#### Titration and ion-exchange

All salts were purchased from Sigma-Aldrich and were of analytical or higher grade. If needed, they were dried and stored over anhydrous calcium chloride. Silver(I) nitrate fixanal was obtained from Sigma-Aldrich. Standard solution used in potentiometric titrations was cross-referenced with a standard solution of sodium chloride on a regular basis.

### Synthesis

#### Equipment

All microwave syntheses were performed in a Discover^®^ SP microwave synthesizer (CEM Corporation) equipped with an Explorer Hybrid utility rack/autosampler. Reflux condensers were always equipped with a drying tube (anhydrous calcium chloride). Reaction mixtures were stirred with the use of magnetic stirrers; smaller magnetic stirbars (higher rpm) are highly advisable when working with silica gels.

#### Compound C1

Solution of 1,3-bis-(3-aminopropyl)tetramethyldisiloxane (5 mmol), 1H-pyrazole-1-carboxamidine hydrochloride (11 mmol) and triethylamine (11 mmol) in anhydrous acetonitrile (15 mL), was prepared in a 50 mL round-bottom flask and heated under reflux for 1.5 h. The resulting, two-phase reaction mixture was allowed to slowly cool and separate for 1 h. The larger, upper layer was then removed and replaced with fresh acetonitrile (10 mL). The contents of the flask were again heated and refluxed for 10 min, after which time the lower layer became waxier and the solvent could easily be decanted. This process was performed twice more and the remaining, resinous product was evaporated under reduced pressure (lyophilization is highly recommended to speed up the removal of residual solvent and reduce the risk of the highly viscous resin bubbling and clogging the flask opening). Yield: 85.21% as pale yellow resin. ^1^H NMR (400 MHz, D_2_O): δ = 0.12 (s, 12H); 0.59 (m, 4H); 1.60 (m, 4H); 3.16 (t, J = 6.9 Hz, 4H). MS (ESI): m/z = 333 [M+H^+^]^+^; 369/371 [M+Cl^-^]^-^; 403/405 [M+HCl+Cl^-^]^-^.

#### Compound C2

A solution of triethylamine (6 mmol), 1,3-bis-(3-aminopropyl)tetramethyldisiloxane (6 mmol) in anhydrous acetonitrile (10 ml) was heated to reflux in a 50 mL, two-necked, round-bottom flask. Arginine methyl ester dihydrochloride (6 mmol) was added to the boiling mixture in small portions over 2.5 h. The stirring was continued for another 30 min. The two-phase mixture was left to separate and cool, and left overnight in the freezer. The upper layer was decanted, the residue was washed twice with acetonitrile (10 mL) and then dissolved in hot absolute ethanol. The solution was evaporated under reduced pressure and lyophilized. Yield: 46.56% as yellow wax. ^1^H NMR (400 MHz, D_2_O): δ = 0.11 (s, 3H); 0.12 (s, 3H); 0.13 (s, 6H); 0.53–0.65 (m, 4H); 1.48–1.71 (m, 8H); 2.90 (t, J = 7.4 Hz, 2H); 3.10–3.29 (m, 4H); 3.35 (t, J = 6.4 Hz, 1H); MS (ESI): m/z = 405 [M+H^+^]^+^; 439/441 [M+Cl^-^]^-^; 475/477 [M+HCl+Cl^-^]^-^.

#### Intermediates I1-I3

To a solution of 1,3-bis(3-aminopropyl)tetramethyldisiloxane (1 mmol) in anhydrous ethyl alcohol (3 mL) in a 10 mL reaction vessel with a small magnetic stirbar, the corresponding isomer of pyridine carboxaldehyde was added (2 mmol). The microwave-assisted synthesis was performed using a two-step program: first, the mixture was heated and held at 50°C for 60 s (50 W) and then ramped to 100°C and irradiated for further 10 min (100 W). Afterwards the mixture was evaporated under reduced pressure, cooled to 0°C and left to solidify in room temperature overnight. **I1**. Yield: 99.43% as white solid. M.p. 75–85°C. ^1^H NMR (400 MHz, CDCl_3_): δ = 0.06 (s, 12H); 0.56 (m, 4H); 1.73 (m, 4H); 3.63 (t, J = 7.1, 1.3 Hz, 4H); 7.58 (dd, J = 4.4, 1.6 Hz, 4H); 8.24 (s, 2H); 8.69 (dd, J = 4.4, 1.6 Hz, 4H). **I2**. Yield: 98.22% as light yellow solid. M.p. 44–46°C. ^1^H NMR (400 MHz, CDCl_3_): δ = 0.07 (s, 12H); 0.55 (m, 4H); 1.75 (m, 4H); 3.62 (td, J = 7.1, 1.2 Hz, 4H); 7.34 (dd, J = 7.9, 4.8 Hz, 2H); 8.11 (dt, J = 7.9, 1.9 Hz, 2H); 8.30 (s, 2H); 8.64 (dd, J = 4.8, 1.7 Hz, 2H); 8.85 (dd, J = 1.5 Hz, 2H). **I3**. Yield: 96.83% as light brown solid. M.p. 33–37°C. ^1^H NMR (400 MHz, CDCl_3_): δ = 0.06 (s, 12H); 0.56 (m, 4H); 1.75 (m, 4H); 3.66 (td, J = 7.1, 1.3 Hz, 4H); 7.30 (ddd, J = 7.5, 4.9, 1.3 Hz, 2H); 7.73 (dddd, J = 8.0, 7.5, 1.8, 0.6 Hz, 2H); 7.98 (dt, J = 7.9, 1.1 Hz, 2H); 8.37 (s, 2H); 8.63 (ddd, J = 4.9, 1.7, 1.0 Hz, 2H). Similar yields were obtained after 80 min reaction in 10 mL of refluxing anhydrous ethanol.

#### Compounds C3-C5

To a refluxing solution of methyl iodide/benzyl bromide (6 mmol) in anhydrous acetonitrile (5 ml) in a 50 mL two-necked, round-bottom flask, a solution of **I1-I3** (1 mmol) in acetonitrile (5 ml) was added in small portions during the course of 5 h, with continuous stirring. The optimal interval between subsequent additions can be estimated at the beginning of the reaction, by observing the time needed for the mixture to stop changing its color (it can turn near-black in appearance at the end). Afterwards, the solution was cooled, evaporated three times under reduced pressure (with dissolving of crude product in 1–2 mL of anhydrous ethanol in-between evaporations) and lyophilized 2–3 times (until no boiling under reduced pressure was observed). Frozen and crushed products were then transferred to and stored in vials filled with argon, inside a desiccator over anhydrous calcium chloride. **C3**. Yield: 93.51% as brownish-red, resinous solid. ^1^H NMR (400 MHz, D_2_O): δ = 0.14 (s, 12H); 0.61 (m, 4H); 1.81 (m, 4H); 3.80 (td, J = 6.7, 1.2 Hz, 4H); 4.43 (s, 6H); 8.26 (d, J = 6.8 Hz, 4H); 8.59 (s, 4H); 8.90 (d, J = 6.6 Hz, 4H). MS (ESI): m/z = 228.1 [M^2+^]^2+^; 583.0 [M^2+^+I^-^]^+^; 836.8 [M^2+^+3I^-^]^-^. **C4**. Yield: 91.94% as dark yellow oil (solidifies into a yellow, waxy solid on standing). ^1^H NMR (400 MHz, CD_3_CN): δ = 0.07 (s, 12H); 0.57 (t, 4H); 1.95 (m, 4H); 3.68 (td, J = 6.9, 1.4 Hz, 4H); 4.37 (s, 6H); 8.07 (dd, J = 7.8, 6.5 Hz, 2H); 8.45 (t, J = 1.4 Hz, 2H); 8.72 (s, 2H); 8.74 (d, J = 5.3 Hz, 2H); 9.10 (s, 2H). MS (ESI): m/z = 228.1 [M^2+^]^2+^; 583.0 [M^2+^+I^-^]^+^; 836.8 [M^2+^+3I^-^]^-^. **C5**. Yield: 90.58% as dark red, waxy solid. ^1^H NMR (400 MHz, CDCl_3_): δ = 0.05 (s, 12H); 0.51 (m, 4H); 1.68 (m, 4H); 3.62 (t, J = 6.6 Hz, 4H); 6.35 (s, 4H); 7.37 (m, 6H); 7.77 (m, 4H); 8.08 (dd, J = 8.1, 6.1 Hz, 2H); 8.59 (s, 2H); 8.87 (dt, J = 8.1, 6.1 Hz, 2H); 9.68 (d, J = 6.1 Hz, 2H); 10.14 (s, 2H). MS (ESI): m/z = 304 [M^2+^]^2+^; 687/689 [M^2+^+Br^-^]^+^; 847/849/851 [M^2+^+3Br^-^]^-^.

#### Materials M1-M2

To a 50 mL round-bottom flask, 1.5 g of propylamine-functionalized silica gel **P2** (equal to 2.6 mmol of propylamine groups), triethylamine (4 mmol) and either 1H-pyrazole-1-carboxamidine hydrochloride (**C1**) or arginine methyl ester dihydrochloride (**C2**) (5.2 mmol each) and 10 mL of anhydrous acetonitrile were added. The mixture was refluxed for 1.5 h and further stirred overnight in room temperature. Afterwards, the material was filtered off, washed with methanol, water, methanol again, diethyl ether and dried. **M1**. Yield: 1.57g of white powder. Calc. modification rate: 43.8%. **M2**. Yield: 1.70 g of pale yellow powder. Calc. modification rate: 57.6%.

#### Materials M3-M5. 1^st^ step

Propylamine-functionalized silica gel **P2** (1.73 g, corresponding to 3 mmol of amine groups), anhydrous ethyl alcohol (3 mL) and the corresponding isomer of pyridine carboxaldehyde (3 mmol) were mixed in a 10 mL reaction vessel with a small magnetic stirbar. The microwave-assisted synthesis was performed using a two-step program: first, the mixture was heated and held at 50°C for 60 s (50 W) and then ramped to 100°C and irradiated for further 20 min (100 W). The silica gel was filtered off, washed with methanol and diethyl ether and dried. The modified materials were used immediately in the second step. **2** ^**nd**^ **step**. To a refluxing solution of methyl iodide/benzyl bromide (4.5 mmol) in anhydrous acetonitrile (5 ml) in a 50 mL two-necked, round-bottom flask, a dispersion of material (obtained in the first step) in acetonitrile (5 ml) was added in small portions during the course of 2 h. The mixture was allowed to cool down, the material was filtered off, washed with methanol and diethyl ether and dried. **M3**. Yield: 1.54 g of orange powder. Calc. modification rate: 47.9%. **M4**. Yield: 1.77 g of bright yellow powder. Calc. modification rate: 41.8%. **M5**. Yield: 1.57 g of dark red powder. Calc. modification rate: 41.9%.

### Potentiometric titrations

#### Equipment

Ion-exchange equilibrations for compounds **C1-C5** were performed in a Sonic-10 (Polsonic) ultrasonic bath. All potentiometric titration experiments were performed using a Titrando 888 unit (Metrohm) equipped with a Micro Ag Titrode (combined Ag-ring electrode for very small solution volumes; pH membrane functions as reference electrode). Standard solution of silver(I) nitrate (0.01M) was used as the titer, and the measurements were carried in 15 mL glass vials, necessarily with rapid stirring (small magnetic stirbar). This setup allows precise measurement of halide content in samples in the range of 0.5–40 μmol. All measurements were repeated three times, with differences below 4% between experimental values. To prevent amine/imine groups from complexing and preventing the precipitation of silver halide, the pH of the medium was brought near 2 using sulfuric acid solution.

#### Modification rates

To test materials **M1-M5** for their halide content (equivalent to the loading of positively-charged groups on their surface) samples that would contain 25 μmol of counter-ions if the modification rate was equal to 100% were weighted in vials, covered with 4 mL of acidified water, and stirred vigorously until all particles became mobile in the liquid. Titration was performed using a monotonic volume addition method (50 μL step). To calculate final modification rates and residual propylamine group loading, materials were treated like mixtures of silica gel with target organic functionalities and original side-chains. In order to calculate rates for materials **M3-M5**, which were produced during a two step-synthesis, it was assumed that all pyridine nitrogen atoms were successfully quaternized (the maximum error with this approach is 4% of modification rate, in the unlikely scenario that all amine groups formed imine bonds in the first step of modification).

#### Ion-exchange experiments: disiloxanes

First, samples of compounds **C1-C5** were tested for their halide content; samples that should contain 25 μmol of counter-ions were weighted in vials and dissolved in 4 mL of acidified water. Titration was performed using a dynamic volume addition method. Experimental values were within 5% margin from values expected and deviations were taken into account when preparing 25 mM solutions (halide concentration) in chloroform (**M1-M2**) or chloroform-acetonitrile (9:1, **M3-M5**). For each anion examined, 0.1 mmol of its finely ground sodium salt (0.05 mmol for bivalent anions) was weighted in a 10 mL flask and covered with 4 mL of compound solution and 1 mL of chloroform. The flasks were closed tightly and sonicated for 20 min at 25°C; resulting mixtures were filtered, washed with chloroform and the filtrate was moved to a larger flask. Solvent was evaporated under reduced pressure, 4 mL of acidified water were added to the flask and the contents were stirred until all residue dissolved; samples of 1 mL were then transported to suitable vials and diluted. Titration was performed using a dynamic volume addition method. Exchange rates were directly calculated from the difference between obtained experimental values and the 25 μmol value, corresponding to a 0% exchange rate.

#### Ion-exchange experiments: materials

**Fig 9:**
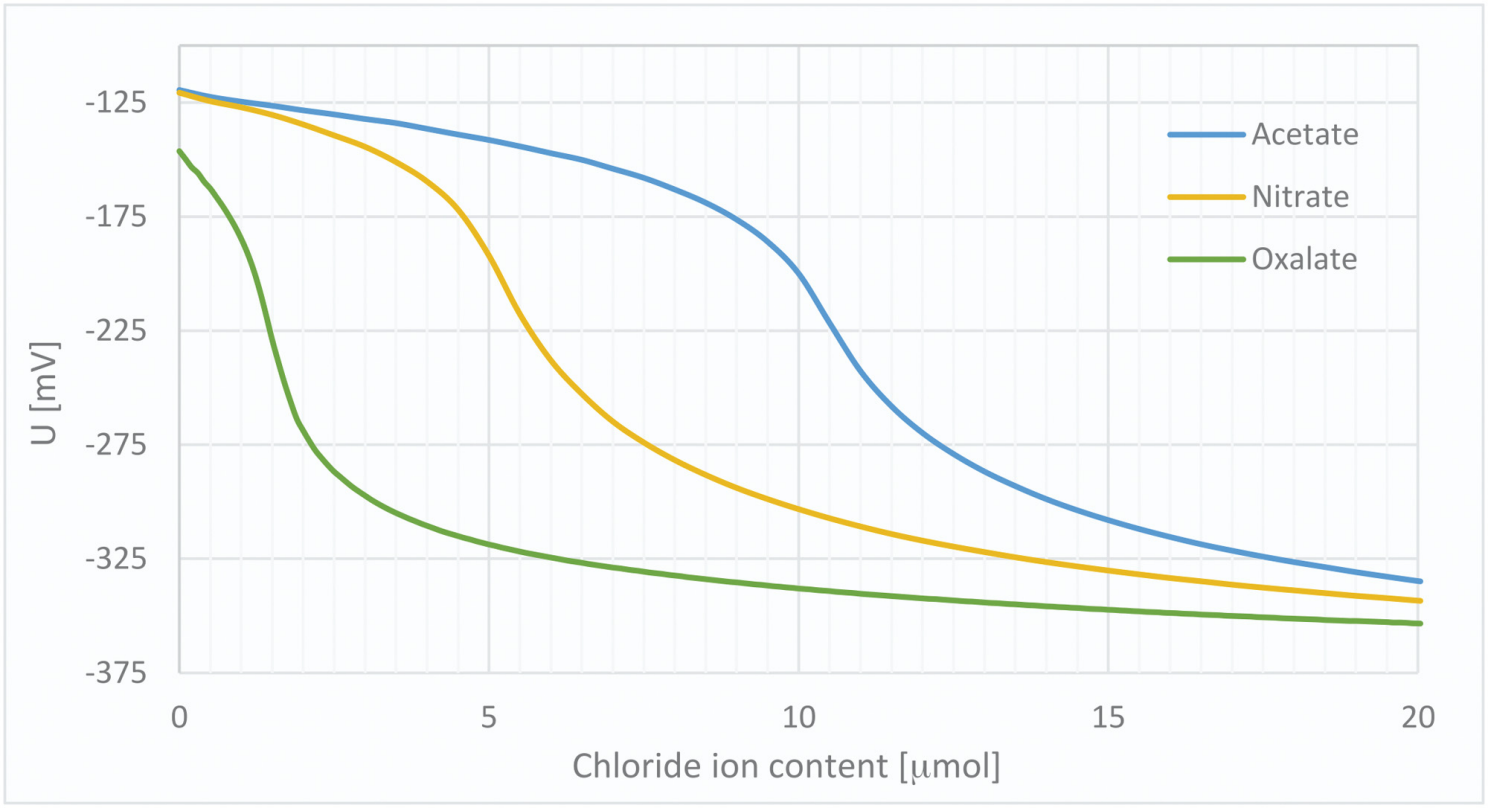
Titration curves obtained for M2 after anion-exchange experiments.

For each anion, 0.1 mmol of its sodium salt (0.05 mmol for bivalent anions) was dissolved in 10 mL of water (**M1-M2**) or anhydrous methanol (**M3-M5**) in a 25 mL flask. To this solution, a material sample containing 0.1 mmol of halide counter-ions was added, and the mixture was stirred rapidly, using a small stirbar, in a closed vessel at 25°C, for 15 min. Afterwards, the material was filtered, washed with methanol and diethyl ether and dried. Recovered silica gel was divided into four equal samples. Titration was performed using a monotonic volume addition method (50 μL step, decreased to 10 μL for samples containing less than 5 μmol of halide). An exemplary set of titration curves obtained for a single material (**M2**) after equilibration with different anions is shown in Fig 9. The actual exchange rates in this case, and amount of different anions present in the material after equilibration, have to be calculated individually using each of the counter-ions’ mass and valency. From these data, it is possible to determine the selectivity coefficients for all possible anion pairs using the equations below:

$$k_{A^{n-}/\;B^{m-}}=\;\frac{{\left[A\right]}_{M}^{m}/\;{\left[B\right]}_{M}^{n}}{{\left[A\right]}_{S}^{m}/\;{\left[B\right]}_{S}^{n}}\;,\;k_{B^{n-}/\;C^{m-}}=\;\frac{k_{A/C}^{n}}{k_{A/B}^{m}}$$

where [A], [B], [C] are anion concentrations, subscripts indicate their phase (S—solution, M—material) and superscripts their valency.

## Conclusions

### Compounds and materials

Five novel disiloxane compounds were obtained with good to excellent yields, very high purity was achieved without the need for chromatographic purification methods. Organosilicon compounds were characterized using NMR, ESI-MS and automated titration methods. The corresponding functionalized silica gels were successfully obtained using the same methodology, under only slightly adjusted experimental conditions, with good modification rates (40–60%). They were characterized using FT-IR and potentiometric titrations methods.

### Ion-exchange properties

Automated potentiometry was employed as an efficient, quantitative analytical method to establish ion-exchange preferences of compounds and materials studied. Among guanidinium dipodands, **C1** was found to be exceptionally selective for nitrate, and among pyridinium dipodands, **C4** exhibited selectivity towards benzoate and ascorbate. These compounds leached the above anions effectively from solid phase into the chloroform phase. Additionally, it was found that **C1** precipitated nitrate selectively from aqueous solutions (calculated solubility of 1.316 g/L). This atypical behavior of a siloxane compound could be explained by formation of larger, more rigid supramolecular aggregates, whose presence is evidenced by more complicated FT-IR spectra of the nitrate salt (in comparison to chloride) and semi-empirical calculations. These data show that a dimeric form (2:4) is far more preferable than a monomer (ΔHOF difference of 226.4 kJ/mol).

Materials with the corresponding organic side-chains showed less defined selectivity. In general, solid-phase extraction favored bivalent anions, while solubilization was more effective for singly charged ions. Pyridinium materials **M3-M5**, though susceptible to hydrolysis, in methanolic solutions bound perchlorate anion with selectivity coefficients one order of magnitude higher than other monovalent anions.

## Supporting Information

S1 File Spectroscopy and semi-empirical methods: details and spectra. (PDF)

S1 Table Material specifications for precursor material P2. (DOCX)
