# Supplementary material for: Disiloxanes and Functionalized Silica Gels: One Route, Two Complementary Outcomes—Guanidinium and Pyridinium Ion-Exchangers
Source: PLoS One. 2015 Dec 29;10(12):e0145680. doi: 10.1371/journal.pone.0145680 (PMC4699886; doi:10.1371/journal.pone.0145680)

S1 File

Supporting Information

for

Disiloxanes and Functionalized Silica Gels: One Route, Two Complementary Outcomes - Guanidinium and Pyridinium Ion-Exchangers

Spectroscopic and semi-empirical methods: details
^{1}H NMR and ESI-MS spectra of compounds **C1**-**C5**

Spectroscopic and semi-empirical methods: details

FT-IR spectroscopy. All samples were measured in 200 mg KBr tabs. The mass of samples was 1 mg per tab. The FT–IR spectra were recorded using a IFS 66/s FT-IR spectrophotometer from Bruker, equipped with an MCT detector (125 scans, resolution 2 cm^{-1}).

NMR spectroscopy. The ^{1}H spectra were recorded on a Bruker DPX-400 spectrometer operating at 400.13 MHz. Typical spectral parameters were used for ^{1}H NMR: spectral width 12 kHz, number of data points 65.5 K, 0.488 Hz per point digital resolution, acquisition time 4.09 s, relaxation delay 1 s, pulse width 7.8 µs, number of scans 16. Chemical shifts were corrected to signal of TMS (0.00) or residual H2O (4.79 in D_2O, **C1**-**C2**). Typical concentration of the samples was 0.1 M. The temperature was maintained and measured with Eurotherm BV-T 2000 to an accuracy of 1 K.

Elemental analysis. Samples were analyzed using Vario EL III (Elementar) elemental analyzer. Three separate measurements were performed for each sample.

Mass spectrometry. The ESI (electrospray ionization) mass spectra were recorded on a Waters/Micromass (Manchester, UK) ZQ mass spectrometer equipped with a Harvard Apparatus syringe pump. The measurements were performed in methanol (**C1**-**C2**) and acetonitrile (**C3**-**C5**) solutions. The samples were infused into the ESI source using a Harvard pump at a rate 20 μdm^3/min. The ESI source potentials were: capillary 3 kV, lens 0.5 kV, extractor 4 kV, and the cone voltage 30 V. The source temperature was 120°C and the desolvation temperature was 300°C. Nitrogen was used as a nebulizing and desolvation gas at a flow rate of 100 and 300 dm^3/h, respectively

Semi-empirical method. Semi-empirical calculations (PM6) of the heat of formation (HOF) and the geometric optimization were performed using the Scigress 2.1.0 program (MO-G Version 1.1, Fujitsu Limited, Tokyo, Japan (2008)). ΔHOF is the difference in energy between the complex and the isolated ligand and anion.

^{1}H NMR and ESI-MS spectra of compounds C1-C5

Fig. 1. ^{1}H NMR spectrum of compound C1 (400 MHz, D_2O).

Fig. 2. ^{1}H NMR spectrum of compound C2 (400 MHz, D_2O).

Fig.3. ^{1}H NMR spectrum of compound C3 (400 MHz, D_2O).

Fig.4. ^{1}H NMR spectrum of compound C4 (400 MHz, CD_3CN).

Fig.5. ^{1}H NMR spectrum of compound C5 (400 MHz, $CDCl_3$).

Fig. 6. ESI-MS spectrum of compound C1.

Fig. 7. ESI-MS spectrum of compound C2.

Fig. 8. ESI-MS spectrum of compound C3 (+).

Fig. 9. ESI-MS spectrum of compound C3 (-).

Fig. 10. ESI-MS spectrum of compound C4 (+).

Fig. 11. ESI-MS spectrum of compound C4 (-).

Fig. 12. ESI-MS spectrum of compound C5.

Fig. 1. ^{1}H NMR spectrum of compound C1 (400 MHz, D_2O).

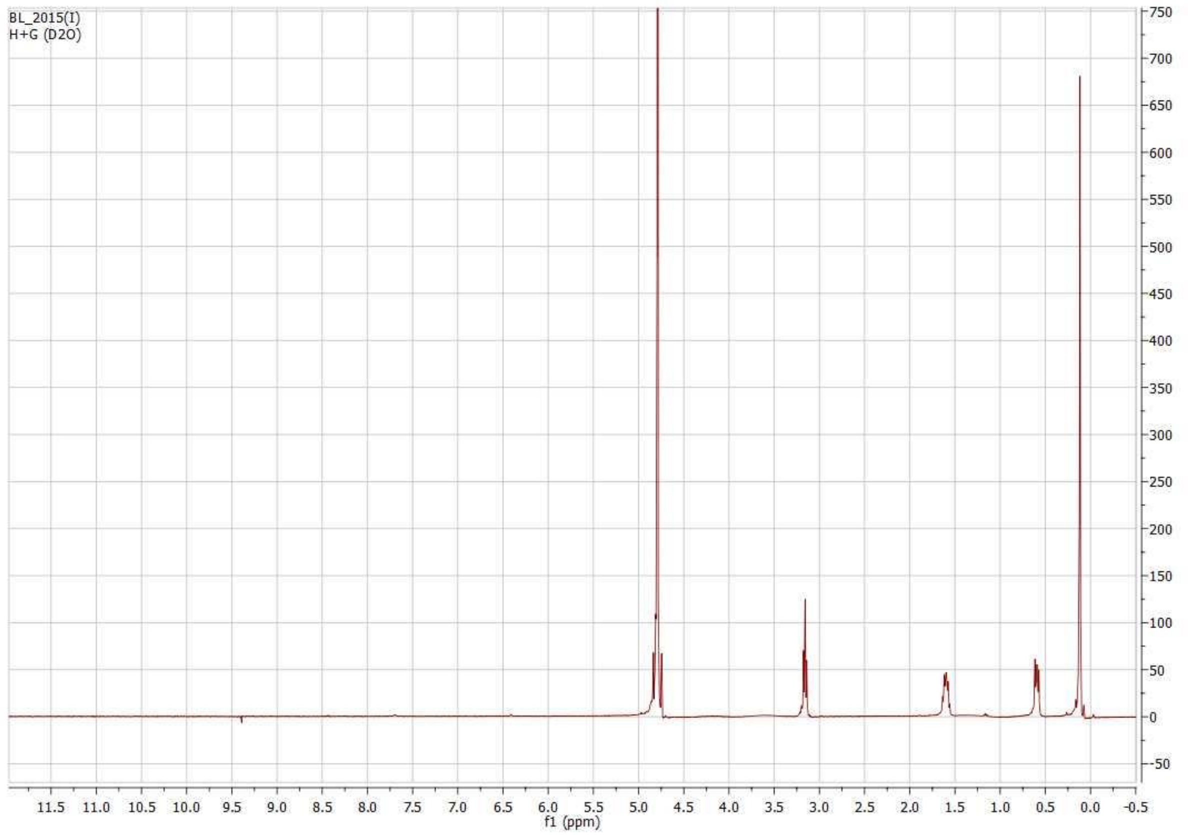

Fig. 2. ^{1}H NMR spectrum of compound C2 (400 MHz, D_2O).

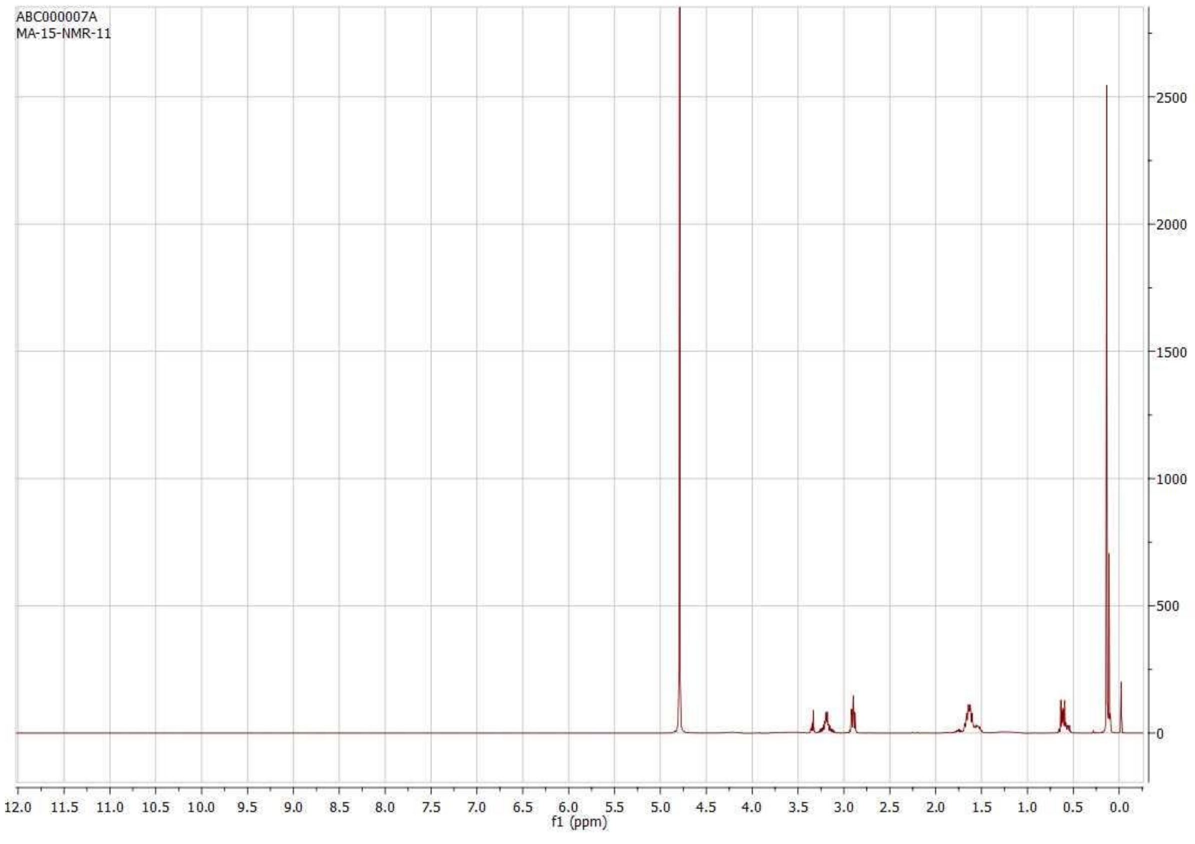

Fig.3. ^{1}H NMR spectrum of compound C3 (400 MHz, D_2O).

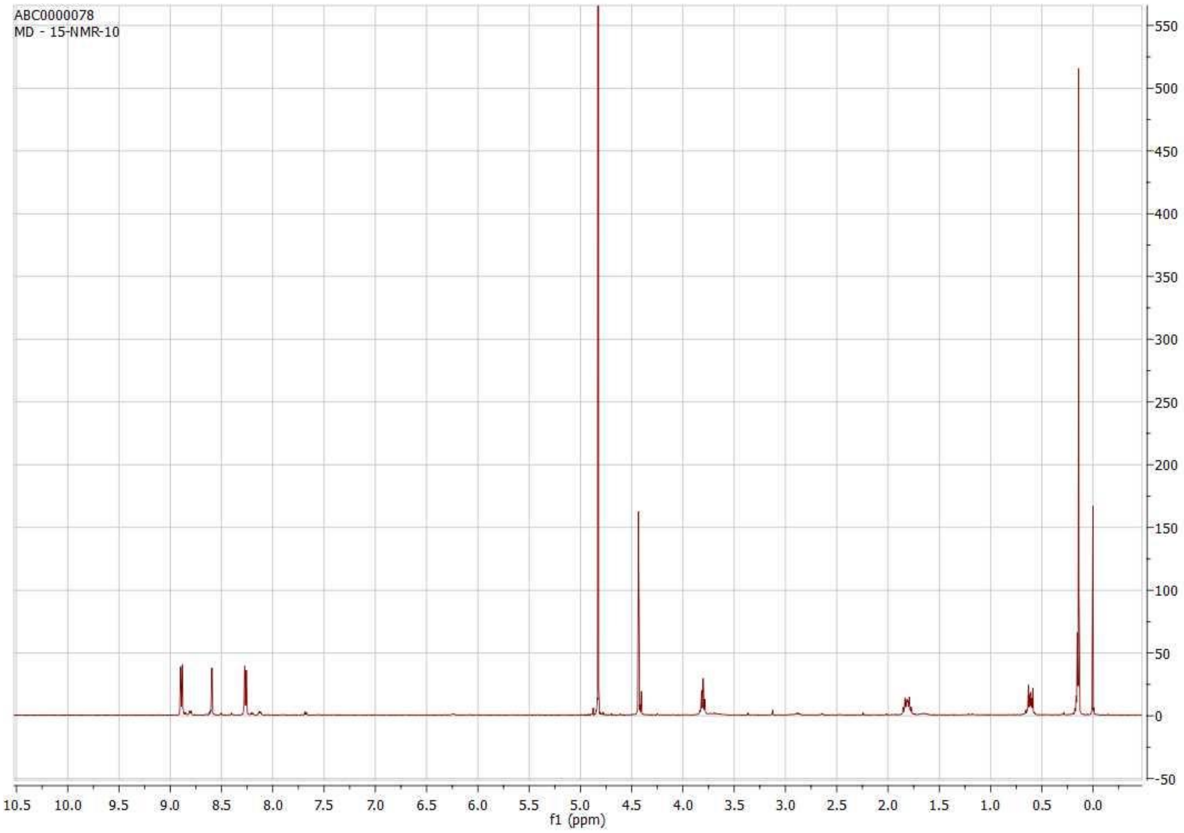

Fig.4. ^{1}H NMR spectrum of compound C4 (400 MHz, CD_3CN).

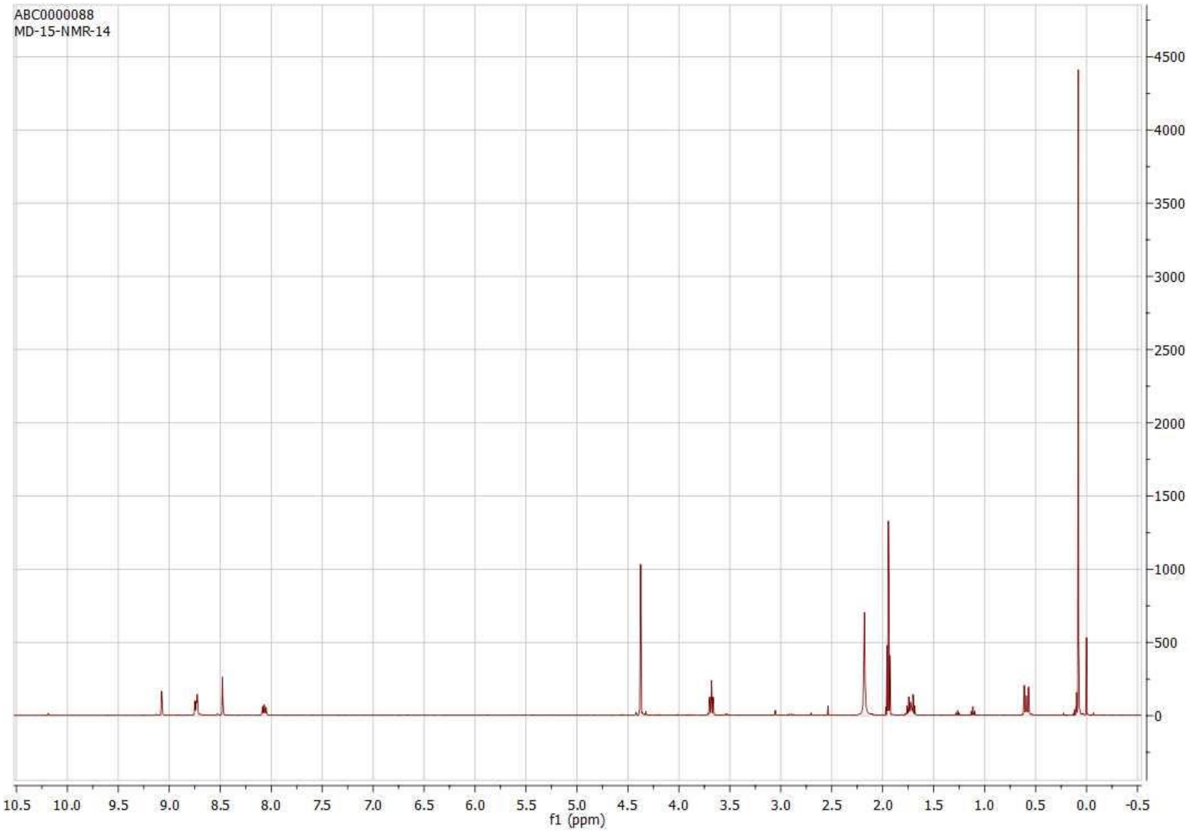

Fig.5. ^{1}H NMR spectrum of compound C5 (400 MHz, $CDCl_3$).

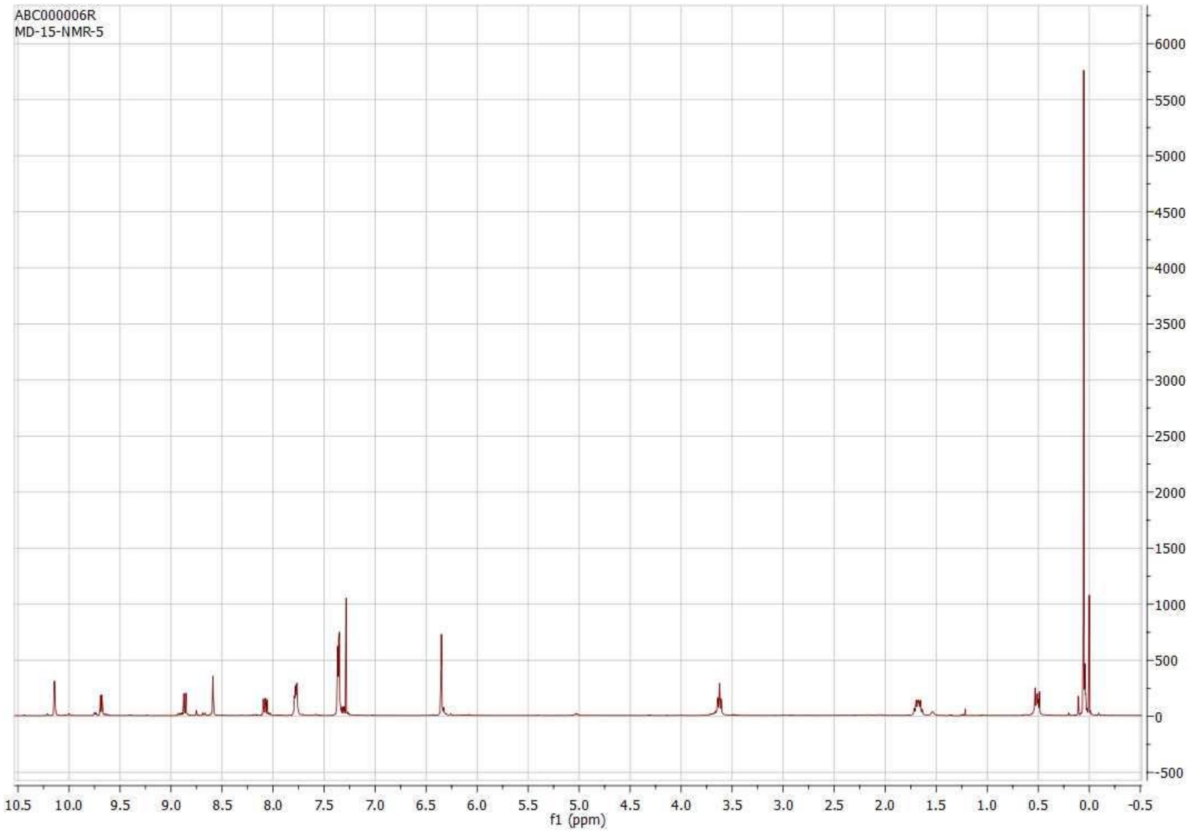

Fig. 6. ESI-MS spectrum of compound C1.

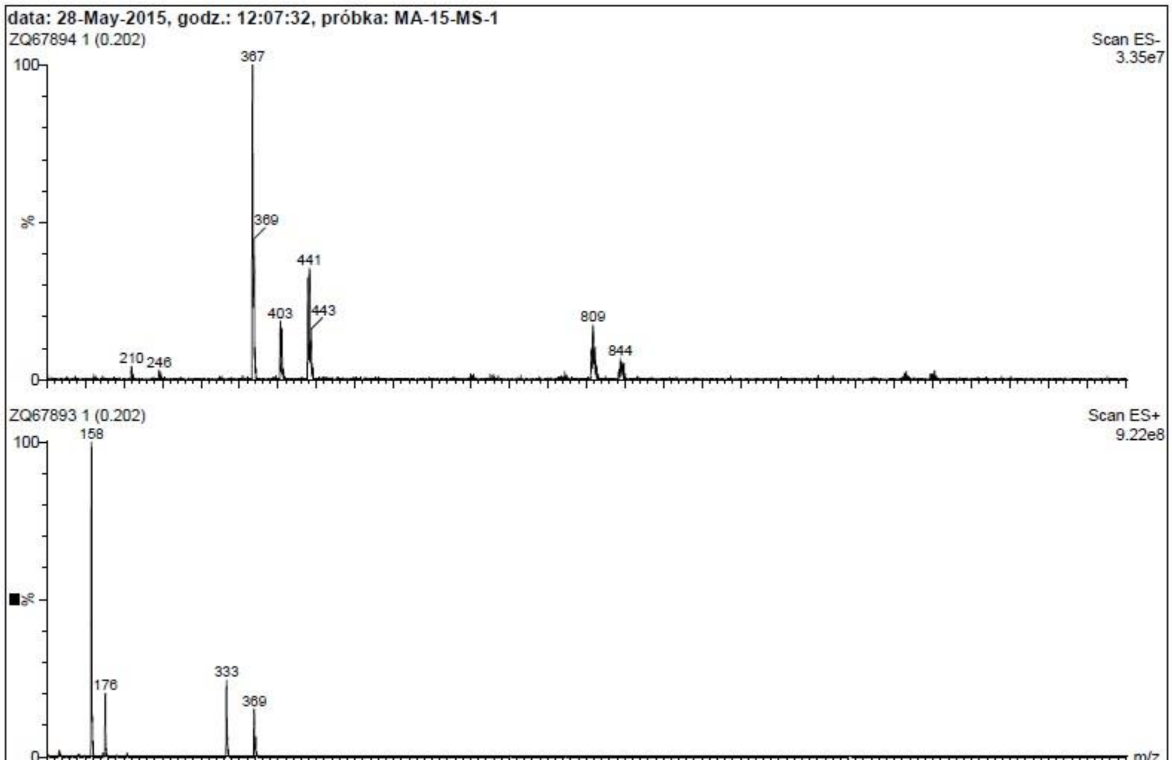

Fig. 7. ESI-MS spectrum of compound C2.

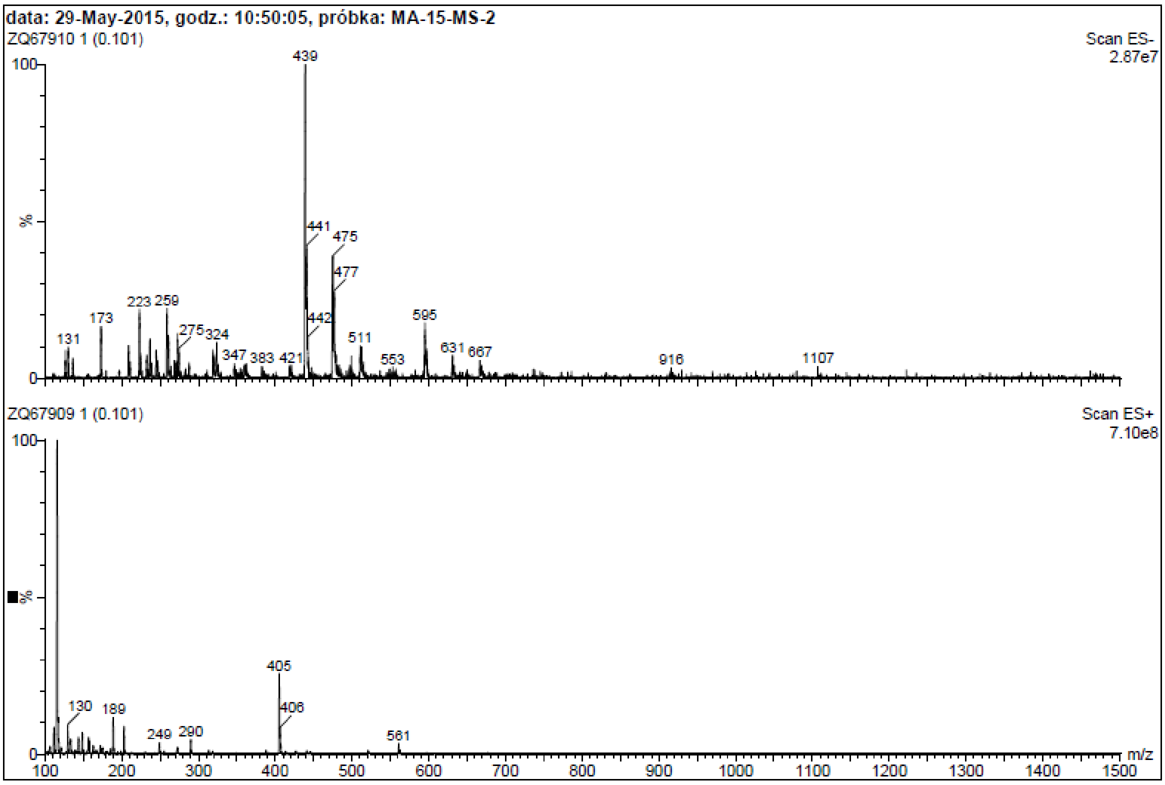

Fig. 8. ESI-MS spectrum of compound C3 (+).

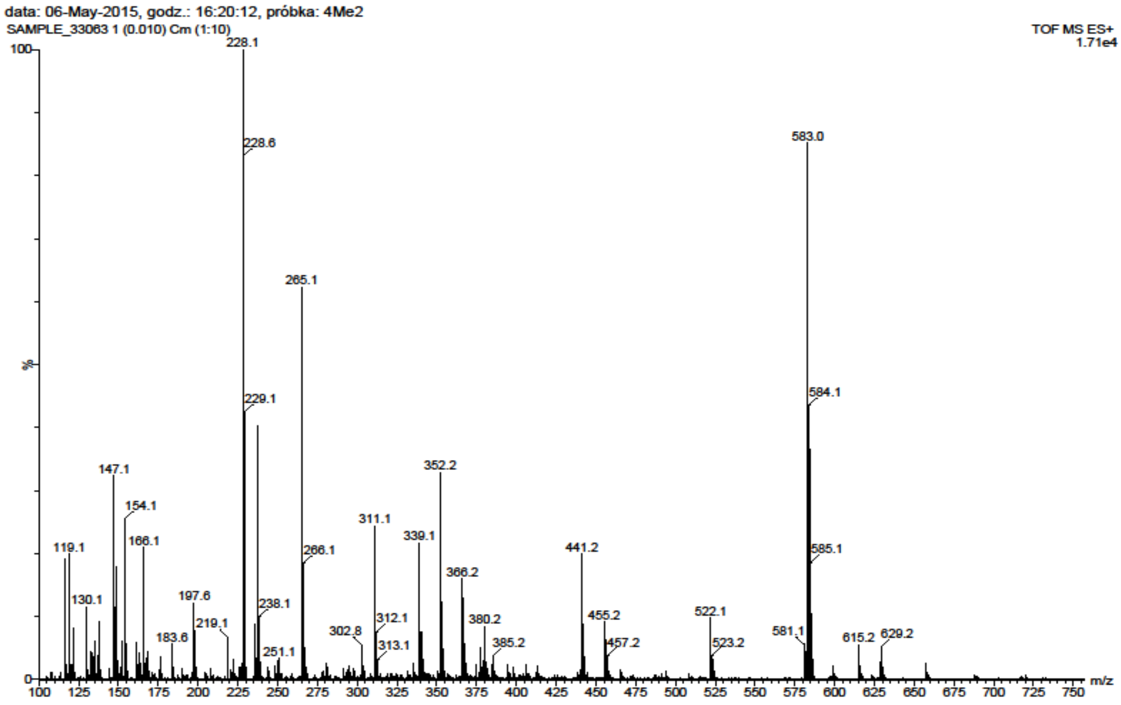

Fig. 9. ESI-MS spectrum of compound C3 (-).

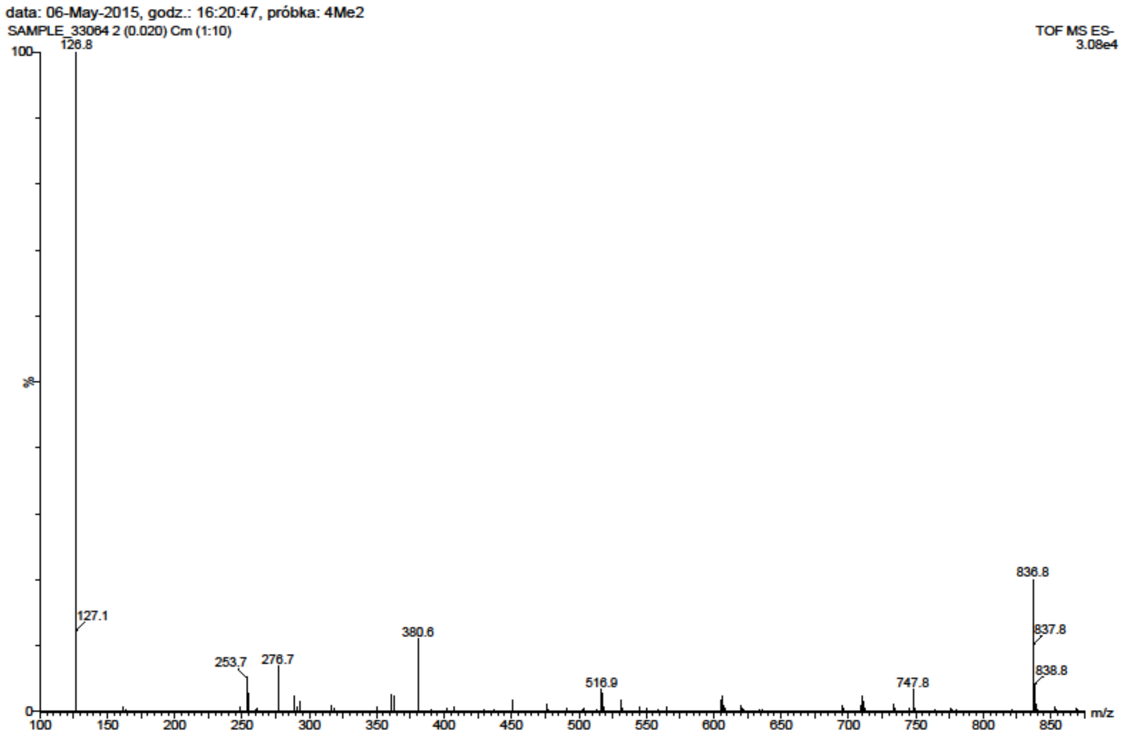

Fig. 10. ESI-MS spectrum of compound C4 (+).

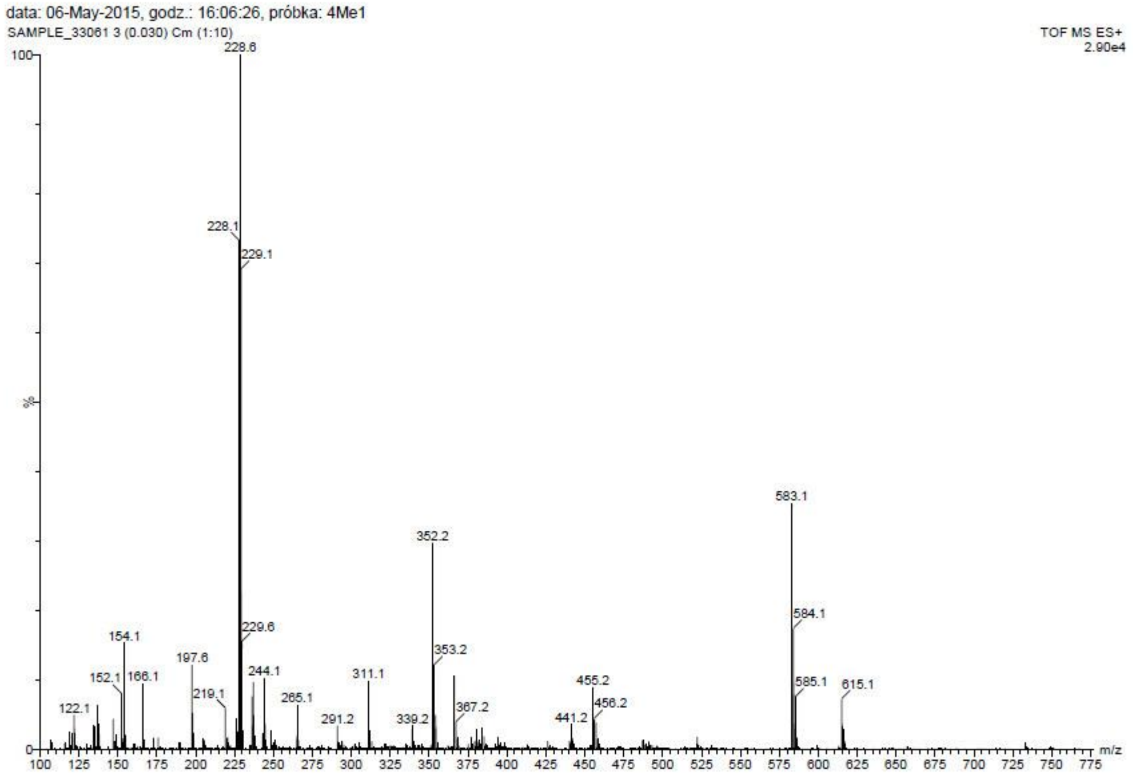

Fig. 11. ESI-MS spectrum of compound C4 (-).

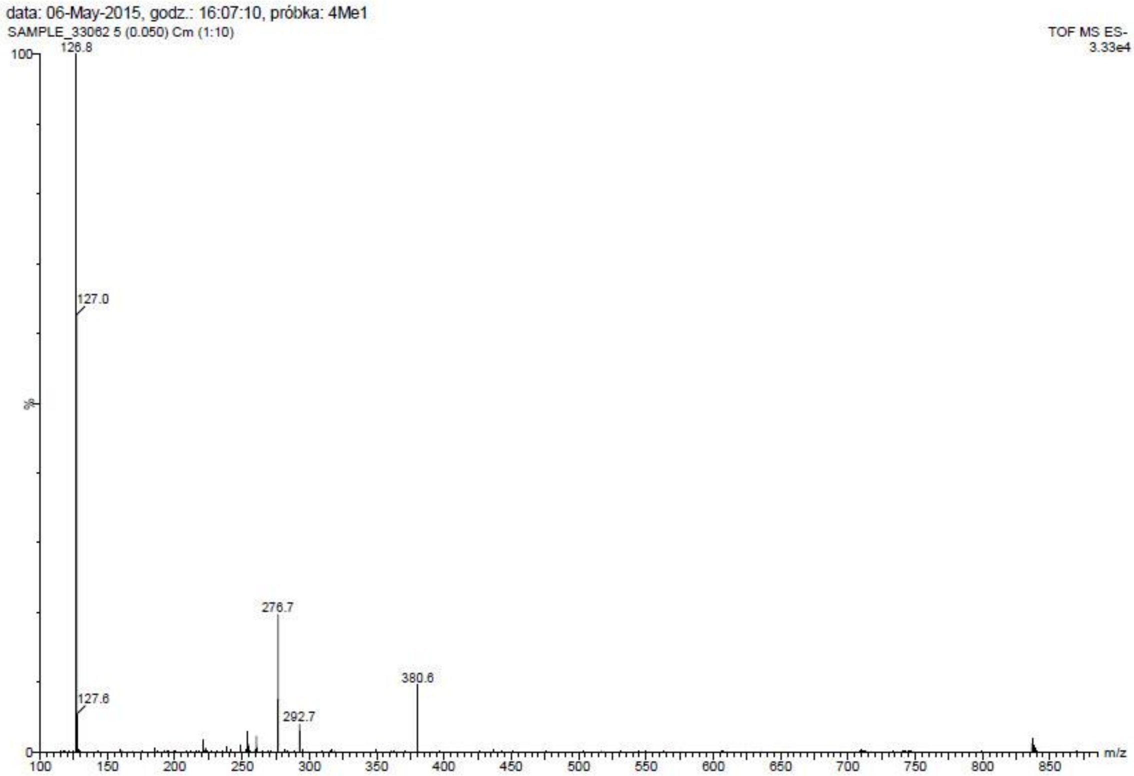

Fig. 12. ESI-MS spectrum of compound C5.

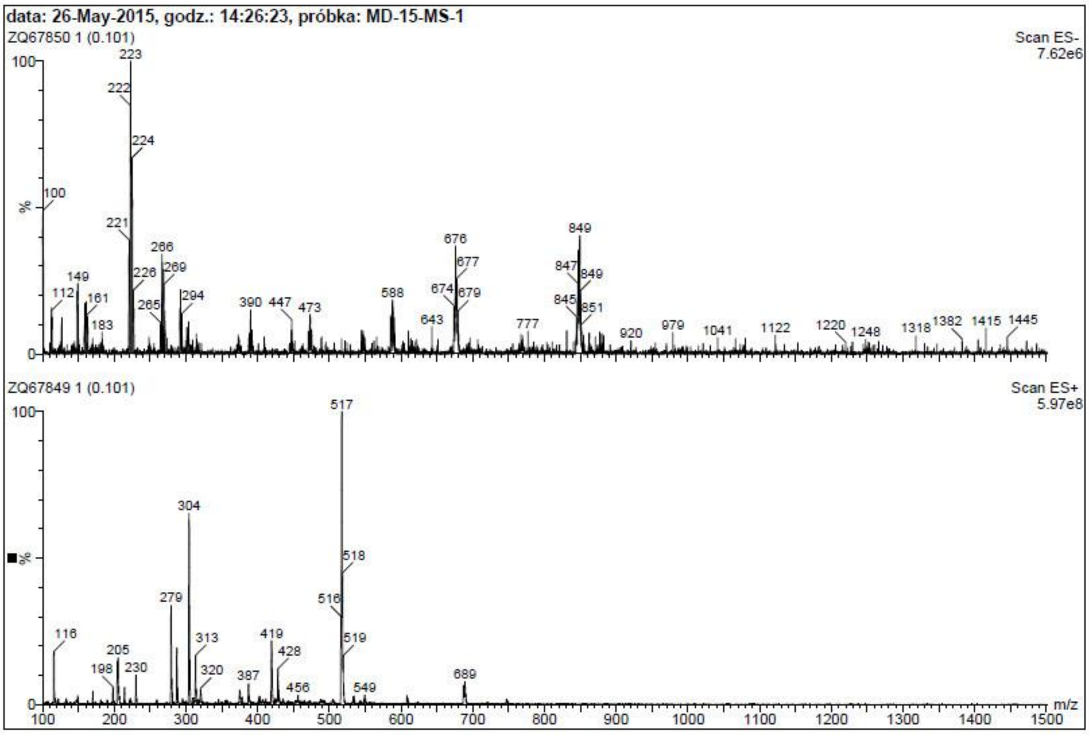

Supplement: S1 File — (PDF) [file pone.0145680.s001.pdf]
