# Supplementary material for: Disiloxanes and Functionalized Silica Gels: One Route, Two Complementary Outcomes—Guanidinium and Pyridinium Ion-Exchangers
Source: PLoS One. 2015 Dec 29;10(12):e0145680. doi: 10.1371/journal.pone.0145680 (PMC4699886; doi:10.1371/journal.pone.0145680)
Supplement: S1 Table — (DOCX) [file pone.0145680.s002.docx]

**S1 Table**. **Specifications of the propylamine-functionalized silica gel precursor P2.**

| **Bare silica specifications** | | | | |
| --- | --- | --- | --- | --- |
| Particle size distribution [μm] | Average pore diameter  [Å, BJH] | | Specific Surface Area  [m2/g, BET] | Specific Pore Volume  [mL/g, BJH] |
| 40 - 63 | 60 | | 470 - 530 | 0.70 – 0.85 |
| **Functionalized silica specifications** | | | | |
| Nitrogen content [%] | Molecular loading [mmol/g] | | Surface coverage  [µmol/m2] | Purity  [%] |
|  | Minimal | Actual^a^ |  |  |
| ≥ 1.68 | 1.20 | 1.73 | ≥ 2.67 | 99.95 |

Full specification sheet and further details are available at <https://www.silicycle.com/>
^a^ Molecular loadings were taken from specifications listed on particular containers and further verified by elemental analysis.
